# A comparison of transcriptomic patterns measured in the skin of Chinese fine and coarse wool sheep breeds

**DOI:** 10.1038/s41598-017-14772-4

**Published:** 2017-10-30

**Authors:** Lichun Zhang, Fuliang Sun, Haiguo Jin, Brian P. Dalrymple, Yang Cao, Tian Wei, Tony Vuocolo, Mingxin Zhang, Qinlin Piao, Aaron B. Ingham

**Affiliations:** 1 Branch of Husbandry, Jilin Academy of Agricultural Science (JAAS), 186 Dong Xinghua st, Gongzhuling, 136100 China; 2 CSIRO Agriculture and Food, Queensland Bioscience Precinct, 306 Carmody Rd, 4067 Queensland, Australia; 3 Department of Veterinary Medicine, College of Agriculture, Yanbian University, Yanji, 133002 China; 4 Institute of Agriculture, The University of Western Australia, Crawley, Perth, Western Australia 6009 Australia

## Abstract

We characterised wool traits, and skin gene expression profiles of fine wool Super Merino (SM) and coarse wool Small Tail Han (STH) sheep. SM sheep had a significantly higher total density of wool follicles, heavier fleeces, finer fibre diameter, and increased crimp frequency, staple length and wool grease (lanolin) production. We found 435 genes were expressed at significantly different levels in the skin of the two breeds (127 genes more highly in SM and 308 genes more highly in STH sheep). Classification of the genes more highly expressed in SM sheep revealed numerous lipid metabolic genes as well as genes encoding keratins, keratin-associated proteins, and wool follicle stem cell markers. In contrast, mammalian epidermal development complex genes and other genes associated with skin cornification and muscle function were more highly expressed in STH sheep. Genes identified in this study may be further evaluated for inclusion in breeding programs, or as targets for therapeutic or genetic interventions, aimed at altering wool quality or yield. Expression of the lipid metabolic genes in the skin of sheep may be used as a novel trait with the potential to alter the content or properties of lanolin or the fleece.

## Introduction

Sheep (*Ovis aries*) are renowned for their ability to produce the natural fibre wool which is an important agricultural commodity used in clothing and textiles. Wool is produced by wool follicles that form across the skin surface *in utero* in a process that is complex and involves many different proteins^1^. The wool fibre is made of terminally differentiated dead keratinocytes which are produced by the wool follicle and has been shown to comprise numerous keratins (KRTs) and keratin-associated proteins (KRTAPs)^2,3^, and an interfilamentous matrix comprised predominantly of KRTAPs, trichohyalin and other proteins^4^. The spatial organization of these proteins and the nature of their chemical bonding in the matrix are thought to largely determine the physical properties of the fibre^5–8^.

The wool follicle is a regenerating mini-organ comprised of the dermal papilla, sebaceous gland, sweat gland and arrector pili muscle^9^ that undergoes a variable cycle of growth (anagen), apoptosis-mediated regression (catagen) and relative quiescence (telogen)^10^. In sheep, this cycle lasts up to two years but this varies greatly between breeds of sheep, and is affected by hormones^11^. Gene expression studies using sequencing, microarray and real time PCR approaches have been undertaken in order to identify signals driving follicle growth and development in different species. In sheep or goat, the gene expression patterns have been explored for genes associated with particular fibre traits, or the expression pattern in different wool follicle cycle phase^12–15^. Some important genes have been associated with traits including fibre diameter^16^, color^17,18^, crimp frequency (or the number of waves per cm of wool fibre length) waves^19,20^, development of secondary follicle^21,22^ or presence of wool follicles^23,24^.

Two breeds of sheep commonly found in China are the Super Merino and the Small Tail Han sheep breeds. The Super Merino (SM) sheep is a new sheep breed approved by the Chinese government in 2014. The breed resulted from crossbreeding Australian male Merino sheep with Chinese Merino, Xinji Fine wool and other local fine wool female sheep in Xinjiang, Jinlin and other localities. SM sheep are known for high quality wool production, with average diameter of the wool ranging from 17.0 to 19.0 µM and average length of wool staples ranges from 8.5 to 11 cm. The Small Tail Han (STH) sheep is an indigenous sheep breed that is well adapted to the local conditions and is renowned for its precocious reproductive ability (fecundity >200%) and wool that is coarser (broad diameter), medullated or heterotypical hair^25^.

In this study, we measured and compared wool morphological phenotypes, hormone levels and transcript profiles between SM sheep and STH sheep in samples collected when the follicles were expected to be in anagen phase. In comparison to STH sheep, we found that the wool of SM sheep was of higher quality, having finer diameter, increased crimp frequency and staple length, as well as being produced from fleeces of increased weight and skin with a greater density of wool follicles. Transcript profiling revealed that genes associated with skin development, lipid/fatty acid metabolism, hair follicle stem cells, such as *FOXI3*, *CD200*, *CD2*4, and fibre shaft structural genes, including *KRTAP1-1* and *KRTAP6-1*, were expressed at higher levels in SM sheep. In contrast, mammalian epidermal development complex (EDC) family genes and other genes associated with skin cornification and muscle function were more highly expressed in STH sheep, consistent with the thickened epidermis and significantly larger primary follicles and increased density of primary follicles, with associated arrector pili muscles, observed in the skin of STH sheep.

## Results

### Characterization of sheep wool and follicle traits

In order, to compare the morphological and production traits of wool between SM and STH sheep, we collected the fleece samples and recorded Greasy fleece weight (GFW), ratio of clean fleece, Fibre Diameter (FD), Staple length (SL), crimp frequency and lanolin content. The results are shown in Table 1 and reveal that significant differences exist between the two breeds for every trait recorded.

**Table 1:** Comparison of wool traits between Super Merino and Small Tail Han sheep.

Wool traits	Super Merino sheep (n = 8)	Small Tail Han) sheep (n = 8)
Greasy fleece weight (kg)	3.67 ± 0.24**	1.43 ± 0.15
Wool yield (%)	56.04 ± 3.58	61.24 ± 3.69*
Staple length (cm)	9.75 ± 1.02**	6.40 ± 0.94
Fibre Diameter (µm)	17.30 ± 0.56	34.18 ± 2.64**
Crimp Frequency n/2.5 cm	13.43 ± 1.21**	4.26 ± 0.57
Lanolin (%)	14.35 ± 2.18**	4.13 ± 0.77

**P < 0.01 and *P < 0.05.

**Figure 1:**
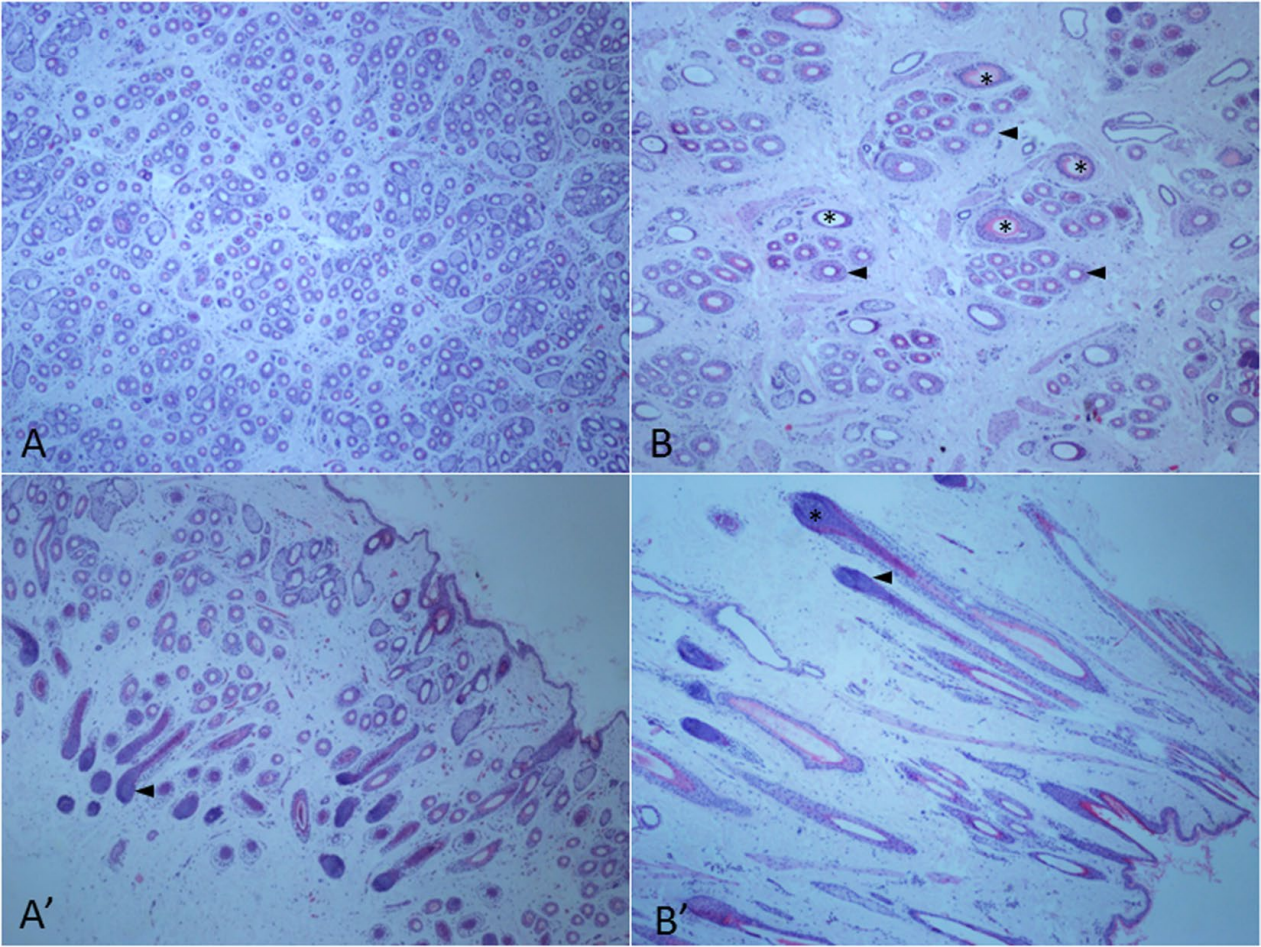
Histological analysis of fixed and hematoxylin-eosin stained skin tissue from Super Merino and Small Tail Han sheep shown at 40 × magnification. Representative primary wool follicles are indicated with stars and arrows indicate the secondary wool follicles. (**A**) Cross section SM sheep. (**A**’) Transverse section of SM sheep. (**B**) Cross section STH sheep. (**B**’) Transverse section of STH sheep.

The skin and follicle morphology was evaluated by microscopy and representative examples are pictured in Fig. 1. As shown in Table 2, the wool follicle density and ratio of secondary to primary follicles were significantly higher in SM sheep. The diameter of dermal papilla and internal root sheath, especially in primary follicles, were smaller in SM sheep than those in STH sheep (Table 2). In transverse section, the skin of STH sheep was more than 250 μm thicker than that of SM sheep, mostly due to a thicker dermal layer. Overall, the wool follicles from the two breeds had similar morphological characteristics indicative of the anagen phase, with the follicle passageway extending from the dermal papilla to the skin, and the dermal papilla and inner root sheath visible and active (Fig. 1).

**Table 2:** Comparison of skin structure and hair follicle traits between Super Merino and Small Tail Han sheep.

traits	Super Merino sheep (n = 8)	Small Tail Han sheep (n = 8)
Skin thickness (µm)	1355.1 ± 91.0	1612.2 ± 73.5**
Epidermal thickness (µm)	18.9 ± 0.3	23.9 ± 0.5
Dermal thickness (µm)	1335.5 ± 90.2	1602.0 ± 64.6
Diameter of primary dermal papilla (µm)	126.4 ± 8.0	141.7 ± 7.6**
Diameter of primary fibre	20.5 ± 0.8	44.5 ± 3.2**
Diameter of secondary dermal papilla (µm)	53.8 ± 2.9	61.0 ± 3.3**
Diameter of secondary fibre	19.4 ± 1.0	35.2 ± 4.7**
Total density of wool follicles (per mm^2^)	69.5 ± 2.9**	31.0 ± 3.1
Density of primary wool follicles (per mm^2^)	3.4 ± 1.3	4.6 ± 1.8
Ratio of secondary wool follicles to primary wool follicles	19.4 ± 3.6**	5.8 ± 3.7

**P < 0.01 and *P < 0.05.

### Quantification of circulating hormone level

**Figure 2:**
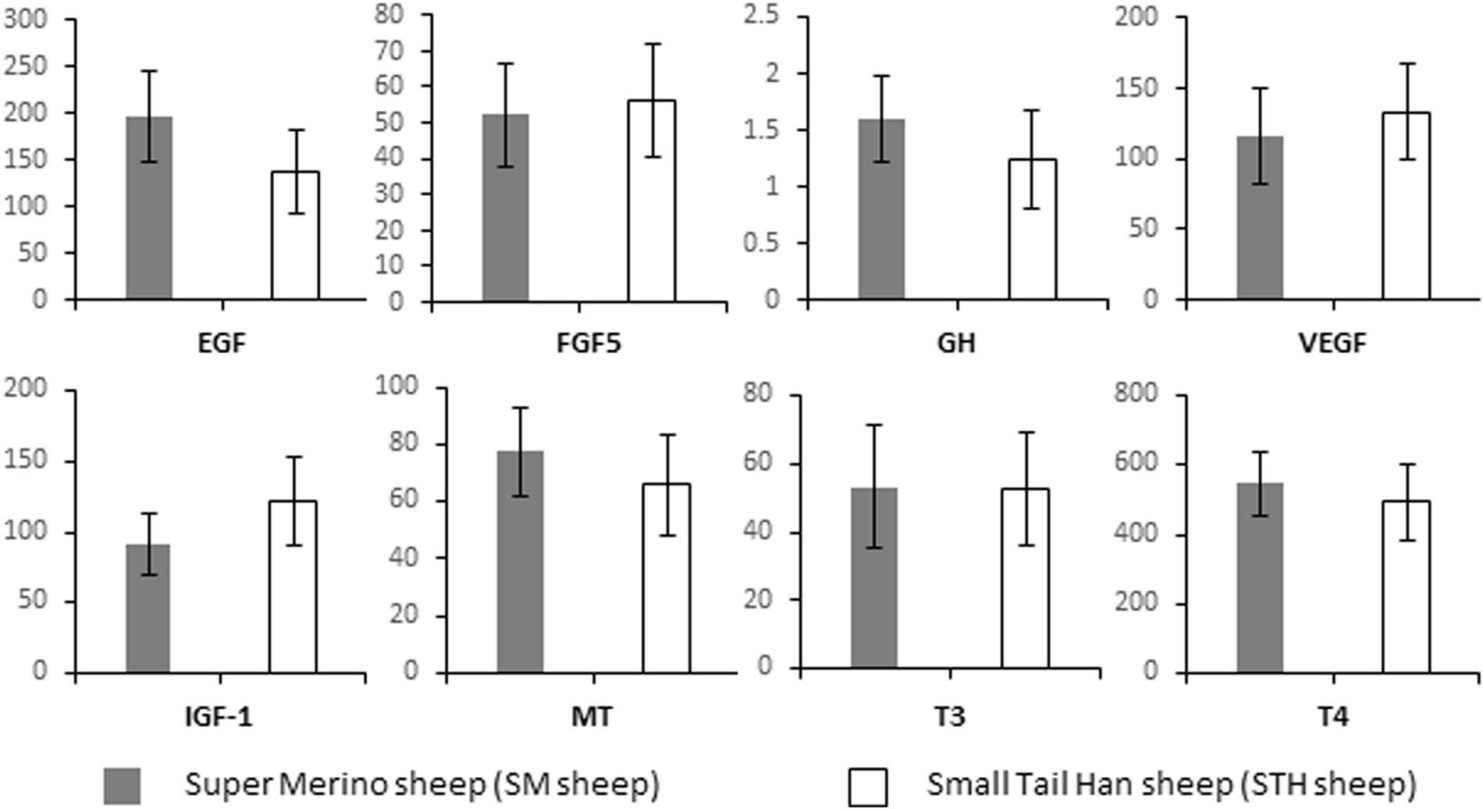
Hormone levels determined by ELISA in blood sampled from Super Merino and Small Tail Han sheep during Autumn. No significant differences were detected between the sheep breeds.

As shown in Fig. 2 no significant differences in the circulating level of Fibroblast growth factor 5 (FGF5), Epidermal growth factor (EGF), Vascular endothelial growth factor A (VEGFA), Insulin-like growth factor 1 (IGF1), Growth hormone (GH), Melatonin (MT), and two thyroid hormones triiodothyronine (T3) and tetraiodothyronine or thyroxine (T4) were determined between SM and STH sheep.

### Transcriptome of sheep skin by RNA sequencing

The map rate of unique reads from the sequenced skin RNA of SM and STH sheep above 0.87. Detailed results of the RNA sequencing and assembly are shown in Table S1. Principal component analysis (PCA) of all mapped genes showed that SM and STH sheep breeds could be distinguished by breed along the axis of the first principal component (Figure S1). After normalization a total 435 differentially expressed genes (DEGs) (|log2^FoldChange^| >0.585 and, false discovery rate [FDR] <0.05) were identified between the groups comprising 127 genes more highly expressed in SM skin and 308 genes in STH sheep skin (Table S2, Figure S2).

Gene ontology analysis on the sets of genes found to be more highly expressed in SM sheep (127 genes) versus those in STH sheep (308 genes) are presented in Tables 3 and 4 respectively. In SM sheep, three sets of genes were identified in multiple classifications. One set of genes was associated with the type I interferon signal pathway, a further set associated with fatty acid/ lipid biosynthesis process, endoplasmic reticulum membrane, lipid particle, cofactor binding and transferase activity and a final set with the cytoskeletal intermediate filament (Table 3). In STH sheep, clusters of genes associated with muscle related GO terms, regulation of DNA and expression in the nucleolus and microtubule organizing, cornified envelope and centrosome were identified (Table 4).

**Table 3:** Gene ontology analysis of genes more highly expressed in Super Merino sheep using the EnrichR program. (p < 0.001).

Gene Ontology Term	p Value	Gene Ontology Classification	Genes
type I interferon signalling (GO:0060337)	5.94E-09	Biological Process	*IFITM3; OAS1; IFI27; OAS2; MX2; MX1; IFI6; ISG15*
lipid biosynthetic process (GO:0008610)	9.08E-05	Biological Process	*SQLE; FA2H; CERS4; ELOVL1; ACSS2; ELOVL4; MBOAT7; ELOVL3; DHCR24; HMGCR; PLB1*
endoplasmic reticulum membrane (GO:0005789)	2.66E-05	Cellular Component	*SQLE; CERS4; ELOVL1; RSAD2; MBOAT7; FKBP1B; ELOVL3; ALG12; FITM2; LRAT; DHCR24; HMGCR*
lipid particle (GO:0005811)	0.000461	Cellular Component	*RSAD2; PLIN4; CIDEA; PLIN2*
intermediate filament (GO:0005882)	0.000269	Cellular Component	*KRTAP4-9; KRTAP9-2; KRT36; KRTAP6-2; KRT79; KRTAP6-1; KRTAP1-1*
cofactor binding (GO:0048037)	9.57E-05	Molecular Function	*SQLE; SDS; ACAD9; ECI2; DHCR24; HMGCR; TKT; CRYM*
transferase activity (GO:0016747)	0.000825	Molecular Function	*CERS4; ELOVL1; AWAT1; MBOAT7; LRAT; DGAT2L6*

**Table 4:** Gene ontology analysis of genes more highly expressed in Short Tail Han sheep using the EnrichR program. (p < 0.001).

Gene Ontology Term	p-Value	Gene Ontology Classification	Genes
muscle system process (GO:0003012)	2.63E-07	Biological process	*DTNA; CCDC78; ROCK1; ROCK2; TPM2; TPM1; PIK3C2A; ADRA1A; ACTG2; ACTA1; PLN; DES; PIK3CA; SLMAP; SCN7A; DMD; MYH11*
regulation of DNA metabolic process (GO:0051052)	0.000941	Biological process	*DNAJC2; CCDC88A; KITLG; RAD50; STAG2; PIK3CA; ESCO1; ATRX; UBE2V2; NBN; PDS5A; SMC3*
contractile fibre (GO:0044449)	2.1E-07	Cellular component	*SYNPO2; TPM2; TPM1; NEXN; NEB; ADRA1A; SYNE2; SYNE1; ACTA1; DES; CFL2; MYH11; DMD; PGM5; SCN1A*
centrosome (GO:0005813)	3.47E-05	Cellular component	*IQCB1; PPP1R12A; ROCK2; CEP350; CEP120; IFT74; MPHOSPH9; NIN; ODF2L; KIF3A; AKAP9; SASS6; CEP162; PKN2; KIF20B; CEP290; PIBF1; LRRCC1*
cornified envelope (GO:0001533)	7.1E-05	Cellular component	*SCEL; CNFN; RPTN; IVL; HRNR*
microtubule organizing (GO:0044450)	0.000441	Cellular component	*NIN; MPHOSPH9; CCDC78; AKAP9; SASS6; CEP162; CEP83; CEP290*
nucleolus (GO:0005730)	0.000657	Cellular component	*ZNF175; RIF1; CEBPE; ANKRD12; PTEN; PSIP1; PHAX; PDS5A; SMC3; HIF1A; SMC4; SYNE1; SENP6; SGOL2; GOLGA4; HSPH1; DNAJB4; CWC22; NBN; RAB11FIP2; ZC3H15; LRRCC1; IQCB1; DIS3; ZNF260; CEP350; ATRX; DNTTIP2; LARP7; PRPF40A; THAP2; UGDH; PM20D2; RAD50; NIN; CREB1; STAG2; ZNF638; PKN2; PPIG; KIF20B; BDP1; CLOCK; BIRC3*
structural constituent of muscle (GO:0008307)	6.09E-07	Molecular function	*PDLIM3; MYBPC2; TPM2; TPM1; NEXN; MYH11; DMD; NEB*

### Analysis of genes associated with sheep skin and wool

A number of the genes that were identified as being more highly expressed in SM sheep were from the keratin (KRT) and keratin associated protein (KRTAP) families, including genes encoding KRT36^26^, KRT79^27^, KRTAP6-1, KRTAP1-1, LOC101114537 (KRTAP4-9L), LOC101115634 (KRTAP9-2) and LOC105605703 (KRTAP6-2L) (Table 5). A further highly expressed gene in SM sheep, *LOC101116068*, encodes keratin-associated protein 9-9-like (KRTAP9-9L). Although this gene was not identified by ontology analysis it is a member of the *KRTAP 9* High/ultrahigh sulphur gene family associated with the hair follicle or fibre shaft^28^. Conversely genes more highly expressed in STH sheep encoded structural proteins of the actin cytoskeleton which compose the muscular cytoskeleton. The actin cytoskeleton genes identified encode ACTA1, PDLIM3, STAG2, PPP1R12A, SYNPO2, CFL2, DMD, NEB, HAP1, IQGAP2 and CD2AP (Table 5).

**Table 5:** Genes associated with cytoskeleton and intermediate filament by GO analysis found to be expressed at different levels in the skin of Super Merino (SM) and Small Tail Han (STH) sheep.

Gene symbol	description	Log2^FoldChange^ (SM vs STH)	FDR	GO term†
*ACTA1*	actin, alpha 1, skeletal muscle	−2.18	5.30E-03	3
*CASP14*	caspase 14	−0.92	4.13E-02	1, 5
*CD2AP*	CD2 associated protein	−0.85	3.13E-02	3, 4
*CFL2*	cofilin 2	−0.82	3.33E-02	3
*DES*	desmin	−1.31	2.08E-06	5
*DMD*	dystrophin	−0.71	2.96E-02	3
*HAP1*	huntington associated protein 1	−1.30	3.95E-02	3
*IQGAP2*	IQ motif containing GTPase activating protein 2	−0.78	2.25E-02	3, 4
*KRT1*	keratin 1	−1.19	1.98E-02	1
*KRT10*	keratin 10	−1.34	1.16E-03	1
*KRT36*	keratin 36	0.80	3.94E-02	2
*KRT79*	keratin 79	0.98	5.04E-03	1, 2
*KRTAP1-1*	keratin associated protein 1-1	1.01	3.49E-02	1, 2
*KRTAP6-1*	keratin associated protein 6-1	1.41	1.27E-02	2,
*LOC101111178*	keratin, type II cytoskeletal 6A-like mRNA	−1.35	9.31E-04	1
*LOC101114537*	keratin-associated protein 4-9-like	1.41	3.86E-02	1, 2
*LOC101115634*	keratin-associated protein 9-2	1.32	3.68E-02	1, 2
*LOC101116068*	keratin-associated protein 9-9-like	1.30	3.86E-02	NG
*LOC105605703*	keratin-associated protein 6-2-like	2.83	6.82E-04	2
*NEB*	nebulin	−1.60	1.27E-02	3
*PDLIM3*	PDZ and LIM domain 3	−0.83	4.27E-02	3
*PKN2*	protein kinase N2	−0.88	2.68E-02	5
*PPP1R12A*	protein phosphatase 1 regulatory subunit 12 A	−0.89	1.39E-02	3
*STAG2*	stromal antigen 2	−0.99	4.42E-02	3, 5
*SYNE2*	spectrin repeat containing nuclear envelope protein 2	−1.09	3.67E-02	5
*SYNPO2*	synaptopodin 2	−0.90	6.22E-03	3
*TPM1*	tropomyosin 1 (alpha)	−0.83	2.51E-02	4
*ZNF175*	zinc finger protein 175	−1.52	5.81E-05	5

^†^GO term: 1. Keratin filament (GO: 0045095); 2. Intermediate filament (GO: 0005882); 3. Actin cytoskeleton (GO: 0015629); 4. Filamentous actin (GO: 0031941); 5. Intermediate filament cytoskeleton (GO: 0045111); NG. Not given.

A search of the full list of identified genes in public databases revealed that only 14 of the 70 epidermal development complex (EDC) family genes were annotated in either of Sheep Genome versions 3.1 or 4.0. In a similar manner, we noted that only 9 of a possible 29 *KRTAP* family members were included in the public annotation of sheep genome version 3.1 and this number actually dropped to just three in genome version 4.0. The result described above is therefore achieved from only a subset of the possible genes. Following a more comprehensive manual search of the genome we were able to identity a total of eight EDC genes that were expressed at different levels in SM and STH sheep (Table 6), with all genes identified having highest expression in STH sheep. No differences in expression were noted between the breeds for the *S100A*, *SPRR2*, *PRD-SPRRII* or *LCE* families. Examination of the clustered sequence reads at the genomic location of putative *KRTAP* genes revealed high numbers of mapped reads. Although we have been unable to incorporate the expression of these genes into a statistical analysis it appears that all of the *KRTAP6* family genes (from *KRTAP6-1* to *KRTAP6-5)* are also likely to be differentially expressed between SM and STH sheep.

**Table 6:** The identification of mammalian epidermal development complex (EDC) family genes and hair follicle stem cells (HFSCs) marker genes in DEGs list.

Gene symbol	description	Log2^FoldChange^ (SM vs STH)	FDR
EDC genes			
* LOC105613519*	late cornified envelope protein 1D-like	−0.92	7.56E-02
* TCHHL1*	trichohyalin like 1	−1.72	1.58E-05
* RPTN*	repetin	−3.41	0
* LOC101120378*	hornerin	−1.14	1.05E-02
* LOC105614079*	filaggrin-like	−1.29	5.50E-03
* LOC105613042*	filaggrin-like	−2.23	1.20E-07
* IVL*	involucrin	−1.05	1.40E-03
* PGLYRP2*	peptidoglycan recognition protein 2	1.44	9.14E-02
HFSCs related			
* FOXI3*	forkhead box I3	1.35	2.62E-03
* FOXJ1*	forkhead box J1	1.75	5.50E-02
* CD200*	CD200 molecule	0.83	3.98E-02
* KRT79*	keratin 79	0.98	5.04E-03
* CD24*	CD24 molecule	−0.69	4.94E-02

### Stem cell associated genes

The *FOXI342, CD200, CD24* and *KRT7943* genes were expressed at significantly different levels between the breeds (Table 6 and Table S2). With the exception of *CD24*, these HFSC marker genes were more highly expressed in SM sheep.

### Pathway analysis

**Figure 3:**
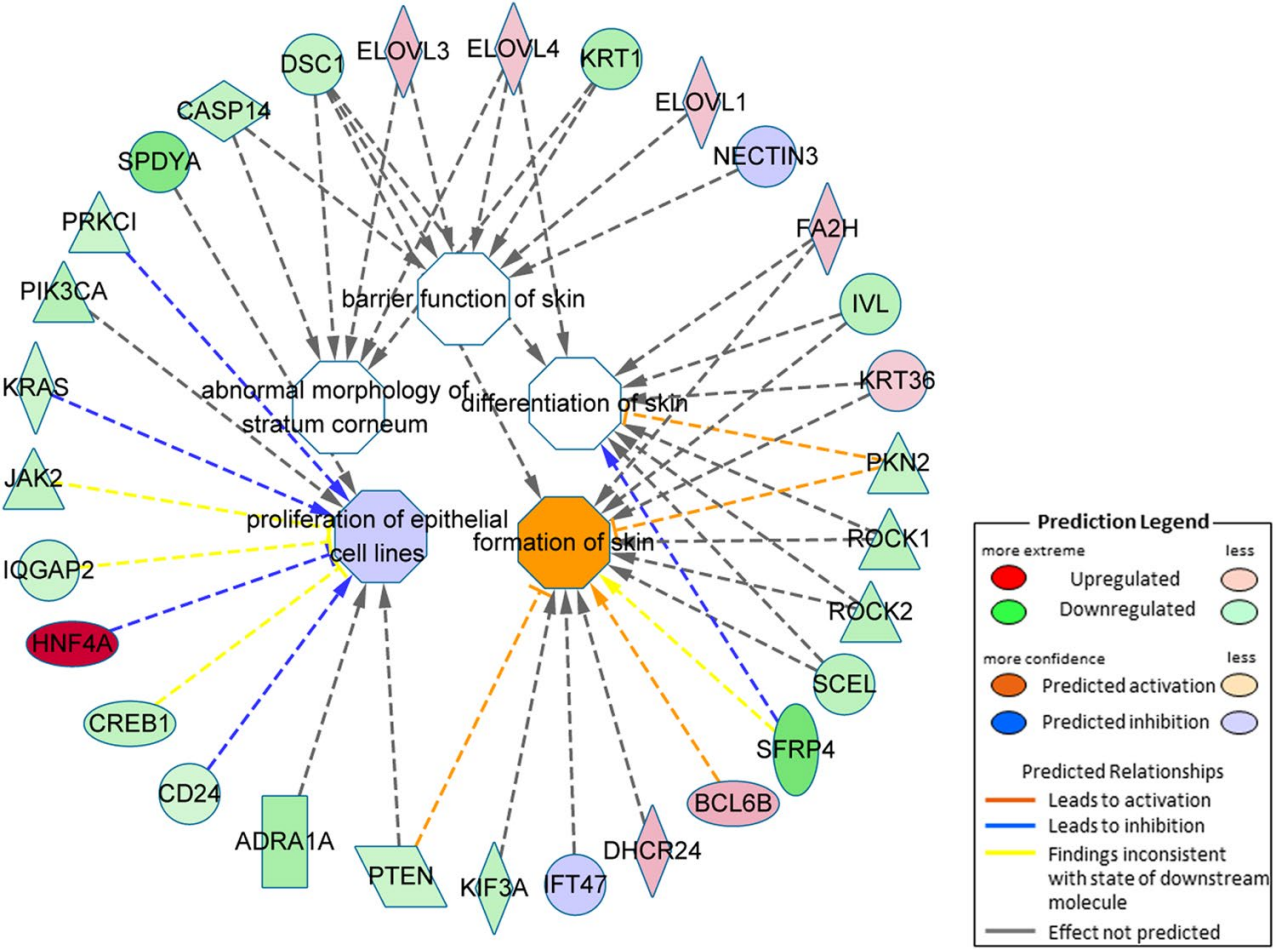
The skin development gene expression correlation network determined by application of IPA methodology. The pathways identified result from a set of genes expressed at higher levels in the Super Merino sheep and shown in green. Genes more highly expressed in Small Tail Han sheep are shown in red.

**Figure 4:**
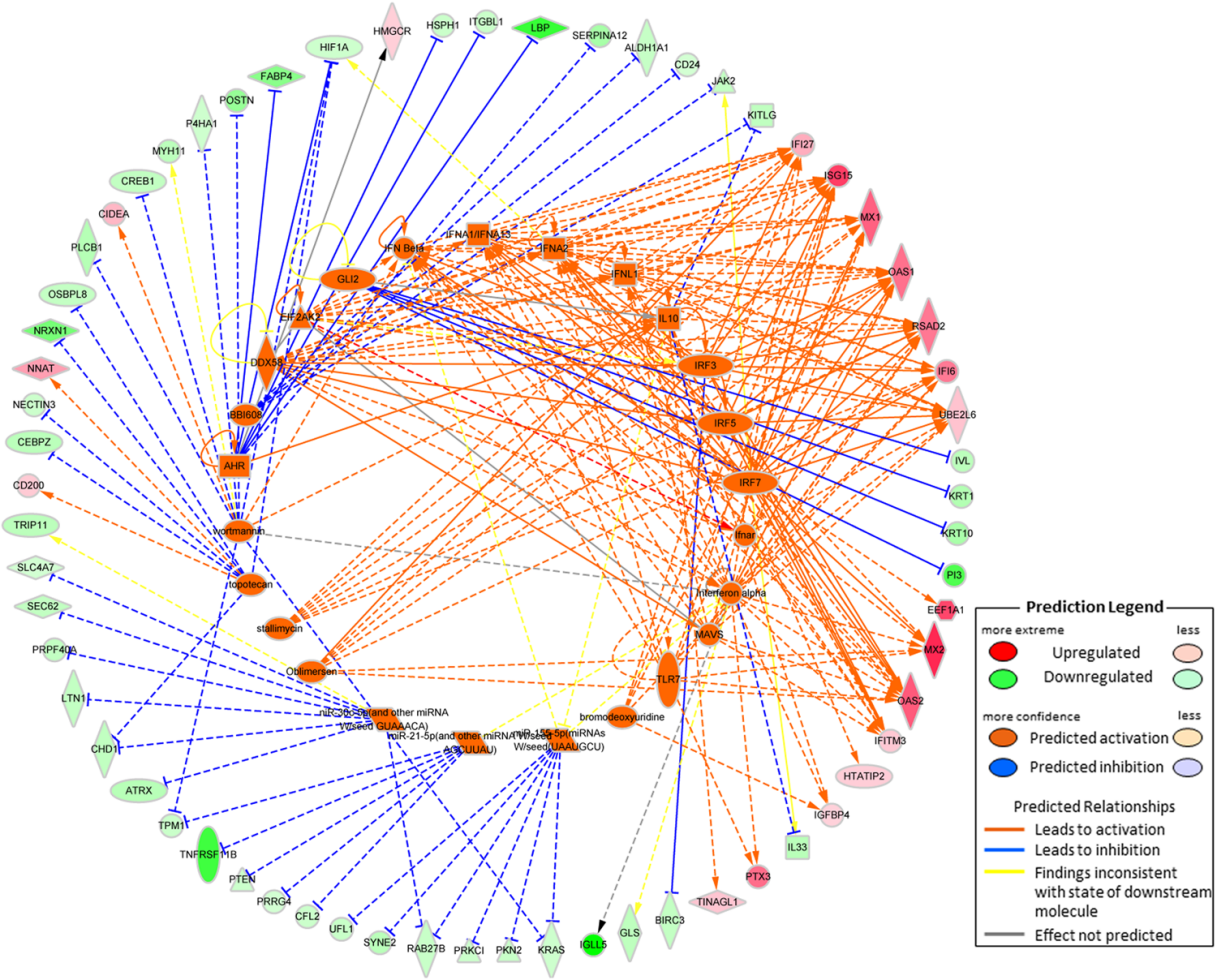
The type I interferon pathway gene network predicted by IPA analysis. Genes shown in green were more highly expressed in the Super Merino sheep. The pathway contained many interferon induced genes which may serve in an antiviral response, including *IFI6*, *IFI27*, *ISG15*, *RSAD2*, *Mx1*, *Mx2*, and *IFITM3*. Genes more highly expressed in STH sheep are shown in red.

**Figure 5:**
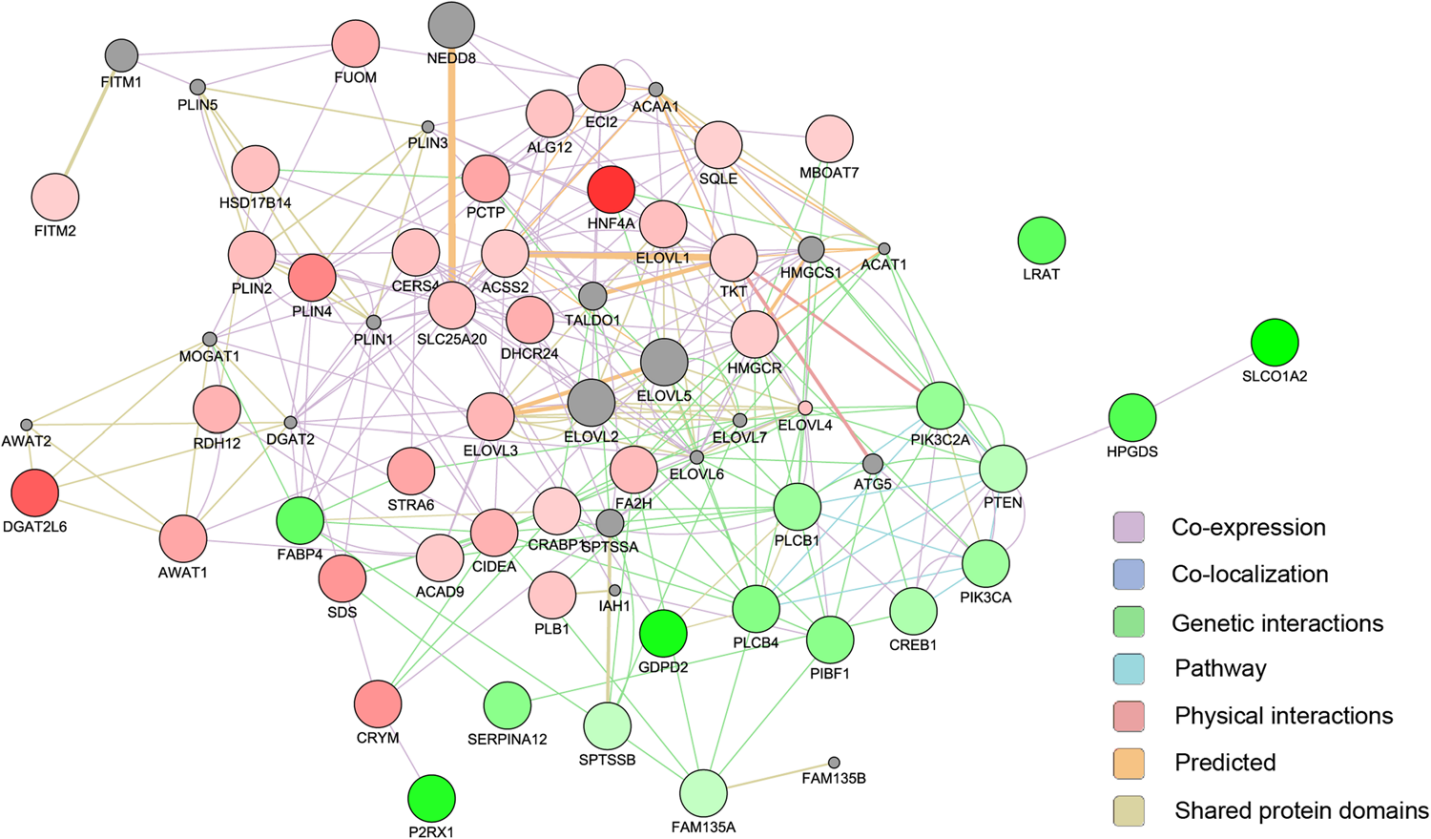
The correlation network of lipid metabolism genes found to be enriched in Super Merino (SM) sheep. Key fatty acid metabolic genes identified include fatty acid synthesis genes (*ELOVL1, 3, 4*), a carnitine carrier involved in transfer of fatty acids to the mitochondrion (*SLC25A20*), and many fatty acid metabolic enzymes (fatty acid 2-hydroxylase gene (*FA2H*), acyl-CoA dehydrogenase family member 9 gene (*ACAD9*), membrane bound O-acyltransferase domain containing 7 gene (*MBOAT7*), and perilipin 2, and 4 genes (*PLIN2* and *4*)). The network also contained genes involved in cholesterol metabolism in SM sheep (Squalene epoxidase gene (*SQLE*) and 3-hydroxy-3-methylglutaryl-CoA reductase gene (*HMGCR*) and the 24-dehydrocholesterol reductase gene (*DHCR24*)), the ceramide synthase 4 gene (*CERS4*) involved in production of a constituent of the integral hair lipid, and genes related to synthesis of triacylglyceride (*STRA6, CRABPI, RDH12, DGAT2L6*) and wax esters (*AWAT1*) respectively.

The genes of three pathways were found to be over-represented in the set of differentially expressed genes. A skin development network involving processes related to the formation, differentiation and proliferation of epithelial cells and the skin barrier was identified in STH sheep (Fig. 3). Genes associated with the type I interferon pathway (Fig. 4) and a complex gene co-expression network based on the set of genes and GO terms associated with lipid metabolism were found to be enriched in SM sheep (Fig. 5).

### Validation of RNA-Sequencing data

**Figure 6:**
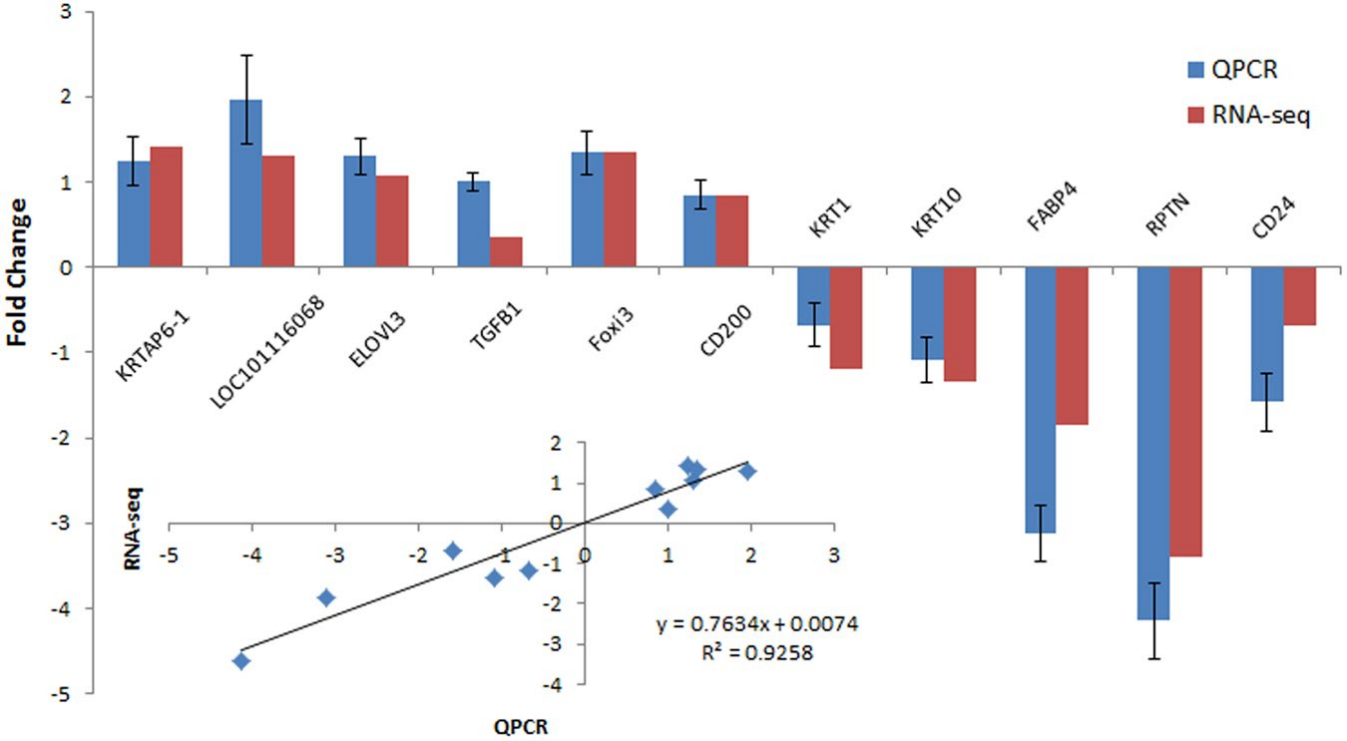
The comparison of transcript expression in terms of fold change as measured by RNA-sequencing and quantitative PCR (qPCR).

Eleven DEGs identified by RNA sequencing were selected for validation by quantitative real time PCR (qPCR). The 2^−ΔΔCt^ method was used to quantify differential expression level during the qPCR analysis and a comparison of results determined via qPCR and RNA-Sequencing are shown in Fig. 6. All selected genes showed similar expression patterns, but the absolute expression levels were not identical between RNA-Sequencing and qPCR. Correlation analysis revealed strong concordance with an R^2^ of 0.9.

## Discussion

In the present study, we have measured a range of wool quality traits in both SM and STH sheep. Large phenotypic differences exist between the two breeds and this provides an opportunity to compare the transcriptome of skin tissue between SM and STH sheep by a high-throughput sequencing method to pinpoint the molecular basis for such differences. An important component of this study was the targeted expression analysis of the gene families that encode the structural proteins of wool and/or contribute to skin barrier function that enabled identification of a further four EDC genes and evidence to suggest that all of the *KRTAP6* family genes (from *KRTAP6-1* to *KRTAP6-5)* were differentially expressed between SM and STH sheep. We determined genes including potential wool follicle stem cell (FSC) markers, components of the wool fibre and lipid metabolic pathways were activated in SM sheep, whereas STH sheep exhibited higher expression of genes associated with cornification of the skin and presence of muscle. With the exception of KRTAP6, none of the candidate DEGs identified here have been previously associated with wool traits in either genetic quantitative trait loci or genome-wide association based studies resulting in many novel candidates for further study^29–33^.

Of the wool fibre structural components, we identified six KRTAP encoding genes in the DEG list (Table 5). Interestingly, all of these genes were more highly expressed in SM sheep. Variation in the sequence of the *KRTAP1-1* gene, which belongs to high-sulphur KAP (HS-KAP) family, has been shown previously to be associated strongly with fibre staple strength and yield in both sheep and goats^34,35^. Here, we found both staple strength and greasy fleece weight (GFW) were significantly higher in SM sheep than in STH sheep. Other differentially expressed KRTAP genes included *KRTAP4-9*, a member of the ultra-high-sulphur protein (UHS-KAP) family that has been shown to be more highly expressed in white hair than black hair (although wool colour of the SM and STH did not differ noticeably by visual inspection)^36^, *LOC101115634* (*KRTAP9-2*) shown to vary in expression level in cashmere goats nor cashmere yield^37,38^ and *KRTAP6-1*, containing abundant polymorphisms^39^, some of which are associated with variation in fibre diameter in sheep^40^. Some KRTAP encoding genes, such as *KRTAP4.9*, *KRTAP6.1* and *KRTAP6.2L*, were upregulated in SM sheep. The products of these genes have been shown to play a critical role in the physico-chemical properties of the wool fibre and may be associated with differences in the crimp of wool observed here between SM and STH sheep^3,41^. Further information on KRTAP family gene expression patterns will likely come to light when the sheep reference genome becomes completely annotated.

Functional FSC are crucial for maintenance of the constant and recurring growth of hair or wool. There are many molecular markers that have been used to identify hair FSCs in different species^42^. Although we did not see the genes encoding classical hair FSCs markers, such as CD34, or KRT15^43^ in the list of differentially expressed genes, we found that *CD200* was increased in SM sheep. CD200 is a common FSC marker in humans^44^, mice^45^ and dogs^46^. As an immune inhibitor, CD200 can attenuate inflammatory response and maintain immune tolerance to hair follicle-associated autoantigens by binding the CD200 receptor (CD200R)^47^. In more general terms CD200 is thought to play a role as a “no danger” signal for the follicle^48^. The high expression of *CD200* in SM sheep may be associated with the higher density of wool follicles. The *FOXI3* gene, a Forkhead family transcription factor, was identified in recent studies as a novel stem cell marker^49,50^. Further, the mutation of *FOXI3* can cause the hairless phenotype in dogs^51–53^. Specific expression of the FOXI3 protein in the epithelium of the hair, whisker placodes and developing teeth has been shown to provide essential signals for the development^54,55^. In respect of morphogenesis and cycling of the hair follicle, expression of *FOXI3* can be thought of as a secondary marker for the hair germ structure^56^. The loss of *FOXI3* results in poor hair regeneration upon hair plucking^56^. In our study, we found the expression of *FOXI3* gene was significantly higher in SM sheep. The KRT79^+^ keratinocytes can migrate out of the reactivated secondary hair germ to form a new hair canal in mouse^27^. Similarly to *FOXI3*, the high expression of *KRT79* indicates secondary hair germ is more active, as evident in more new wool fibres initiated in SM sheep*. CD24* was thought to be a keratinocyte differentiation marker because of its specific expression in the bulge, ORS and the glabrous epidermis^57^. However, *CD24* was not expressed in bulge stem cells, but in the non-bulge inner ORS, and can be looked at as a negative selection marker for HFSCs^44,58^. We also identified the *CD24* gene in the DEG list. It is interesting that the expression of *CD24* was lower in the SM sheep given its potential role in keratinocyte differentiation and the expected higher incidence of differentiation in the follicle rich SM skin. High expression of *CD200* and *FOXI3* and low expression of *CD24* in SM sheep suggests the activation of HFSC is higher in SM sheep than in STH sheep. These factors might all contribute to the high quality wool characteristics of the SM sheep. Based on evidence obtained in other studies, and the information revealed here, we postulate that *CD200*, *FOXI3* and *CD24* might also be useful for the study of wool FSC function in sheep.

The high expression of muscle process genes in STH sheep and the smooth muscle myosin II heavy chain gene, *MYH11* ^59^, was an unexpected finding because STH sheep do not obviously have increased muscle tissue in the epidermis or dermis of skin (Fig. 1). However, after a more considered examination we believe one possible explanation for this result is the presence of higher numbers of primary follicles per unit area in STH skin (Table 2). Whilst the total follicle density is much higher in SM sheep, STH sheep have a higher density of primary follicles. Only the primary follicles have an arrector pili muscle (APM) meaning the STH sheep with more primary follicles per unit area are predicted to have more accompanying APM and with larger primary follicles may have increased APM size per follicle thereby the associated increased muscle gene expression signal detected here (Table 4). Besides muscle genes, two genes encoding keratins associated with epidermal keratinocytes, *KRT1* and *KRT10* were identified with higher expression in STH sheep (Table 5).

The content of lanolin was significantly higher in the fleeces of SM sheep (Table 1) and the concomitant expression of lipid metabolic genes was higher in the skin (Tables 3, S2 and Fig. 5). Lanolin, or wool grease, is secreted by the wool follicle’s sebaceous gland and coats and softens the wool fibre, protecting both skin and fleece from exposure to the elements by assisting in the shedding of water from the fleece^60^. The DEGs, including *DGAT2L6*, *AWAT1*, *CIDEA* and the *PLIN* family members are likely to be expressed in the sebaceous gland^61^ and may be key factors in determining lanolin content of wool. As such, expression level of these genes might serve as novel traits that can be used to select for wool with altered lanolin content or lanolin with altered properties. The increased content of lanolin associated with increased fibre density is clear however it is unclear if the elevated expression of lanolin is essential for fleece health and quality or if it is an artefact of selection for higher follicle density (resulting in increased presence of the accompanying sebaceous gland). The candidate genes identified here might therefore serve as a means for determining if selection for high fibre density can be achieved with reduced lanolin content. Although evidence does exist that variable expression of some of the DEG can affect hair integrity suggesting that limits to this approach are likely. For example, some of the DEGs have been associated with Lichen planopilaris (LPP) characterized with patchy hair loss, perifollicular erythema, perifollicular spines and scarring^62^, hair growth defects^63^. Finally, the lipid metabolic genes might also influence localised energy production in the skin through the product of solute carrier family 25 member 20 gene (SLC25A20), a carnitine carrier that transfers fatty acids to the mitochondrion^64^ providing a further novel target for breed improvement.

## Conclusion

In summary, we have identified differences in wool and skin traits and skin transcriptome profiles between SM and STH sheep. The differences in the transcriptome profiles between the two breeds were consistent with the observed differences in morphological and productivity traits. Many of these genes have not been previously associated with wool characteristics, yield or quality making them novel candidates for use in sheep breeding programs. Indeed expression of the differentially expressed lipid metabolic genes in the skin of sheep may be used as a novel trait with the potential to alter the content or properties of lanolin or the fleece.

## Materials and Methods

### Ethics statement

This study was carried out in strict accordance to relevant guidelines and regulations by the Ministry of Agriculture of the People’s Republic of China. All experimental protocols were approved by the Laboratory Animal centre of Jilin University (SKXK 2015-0006).

### Animals

Eight six-month-old ewes from Super Merino (SM) and Small Tailed Han (STH) sheep breeds respectively were selected from Jinlin province. In order to eliminate the environment effects, two groups of ewes were housed on the same farm with the same complete formula feed and hay. All skin and samples were collected during mid-October when the wool cycle is expected to be in anagen phase.

### Sample collection, preparation, histological examination and endocrine analysis

The wool located 10 cm behind the shoulder blade was shaved and sterilized by 70% alcohol. For each animal two adjacent skin samples were removed using surgical scissors. One sample was immersed into RNAlater reagent (Qiagen, Germany) immediately to avoid RNA degradation and stored at −85 °C for later RNA extraction. The other skin tissue was fixed by 4% paraformaldehyde for histological examination. Transverse and cross sections of the fixed and paraffin embedded samples were stained with hematoxylin-eosin and evaluated by light microscopy.

At the same time, peripheral blood serum samples were collected and the level of circulating hormones, including fibroblast growth factor 5 (FGF5), epidermal growth factor (EGF), vascular endothelial growth factor A (VEGFA), insulin growth factor 1 (IGF1), growth factor (GF), melatonin (MT), thyroxine (T4) and triiodothyronine (T3) were measured by ELISA kits (NeoBioLab, USA). The wool samples from the same ewes were collected during May 2014 and greasy fleece weight (GFW), wool yield, fibre diameter (FD), staple length (SL), crimp and lanolin were measured according to methods described in^65^.

### The cDNA library construction and sequencing

The skin samples were ground in liquid nitrogen with a mortar and pestle. Total RNA was purified using a Trizol Reagent (Life technologies, USA), according to the manufacturer’s instructions. The RNA quality was evaluated using the RNA Integrity Number (RIN) value by Agilent 2100 Bioanalyzer (Agilent, USA). The mRNA was purified, fragmented, and converted into cDNA, adapters were ligated to the end of double-stranded cDNA and the library was created by PCR using the Illumina Truseq RNA Sample Preparation Kit (Illumina, USA) manufacturer’s protocols. Six independent paired-end libraries were sequenced on a HiSeq. 2000 system at the Beijing Genomics Institute (BGI)-Shenzhen, Shenzhen, China (http://www.genomics.cn/index.php) according to manufacturer instructions (Illumina, San Diego, CA, USA).

### Data analyses

RNA sequencing raw reads files (fastq) were checked for quality using FastQC, and were mapped to the *Ovis aries* genome (Oar_v4.0) using advanced RNA-seq plugin on the CLC Genomics Workbench (version 9.0.1, CLC bio, Aarhus, Denmark) with default parameters. The raw counts of each gene was determined using the default Generalized Linear Model (GLM) and Principal Component Analysis was also performed within the advanced RNA-seq plugin. Curves of best fit were used to generate expression values based on read counts following a Negative Binomial distribution. The differentially expressed genes (DEGs) were investigated in the advanced RNA-seq plugin using the EdgeR algorithm (|log2 FoldChange| >0.585 and false discovery rate (FDR) <0.05). Gene ontology enrichment analysis was carried out using EnrichR^66^. The pathway and network was generated using Ingenuity Pathway Analysis (IPA)^67^ and GeneMANIA plugin in Cytoscape^68^. The canonical pathways were identified using thresholds of score (>2) and FDR (<0.05).

### Targeted analysis of skin and wool genes

To determine if the gene expression results were complete we tested for the presence of key candidate genes associated with wool and skin structure in the set of genes annotated in ovine genomes version 3.1 and 4.0. For each genome version the full gene set of gene IDs was exported from CLC bio into a Microsoft Excel spreadsheet. The spreadsheet was then manually searched for the presence of each candidate gene using the Find function. Key candidates included the members of the keratin associated protein (KRTAP) family as well the mammalian epidermal development complex (EDC) family. Recent reports have confirmed expression of at least 29 *KRTAP* genes in sheep and the genes are located in clusters on ovine chromosomes 1, 11, and 21^3,41^. The EDC is a cluster of approximately 70 genes found on ovine chromosome 1 that plays an important role in development of keratinized epidermal structures, such as the rumen, skin, and wool^69^, and has been characterised by our laboratory in an earlier study^61^. The putative expression level of any gene found to be missing from the genome annotation was then determined using the detailed gene chromosomal locations reported in the reference paper^3^. In some instances this location was given an unidentified ID (eg *LOC105613519*) and a likely identity could then be attributed. For the remaining genes it was possible to examine the number of raw reads mapped to each location, although it was not possible to determine statistical significance of these results as they were not included in the original CLC bio gene expression analysis.

### Quantitative real-time PCR (qPCR)

In order to verify the RNA-seq results, a set of ten eleven candidate genes was selected from the list of DEGs and expression level of each was evaluated using quantitative real time PCR (QPCR). Reactions were performed for each individual animal using 1.0 µg of residual RNA from the original extractions described above. The first-strand cDNA was synthesized by TransScript First-Strand cDNA Synthesis SuperMix (Transgen, China) and the QRT-PCR was performed using Takara SYBR® *Premix Ex Taq*™ II (TAKARA, China). The amplification conditions were 95 °C for 10 min of initial stage, followed by 40 cycles of 95 °C for 10 s and 62 °C for 30 s and performed on Bio-Rad CFX96™ Real-Time PCR Detection Systems (Bio-Rad, USA). The reactions of all genes were run on one plate in triplicate for each biological replicate. Relative expression values were calculated by the 2^−ΔΔCt^ method.

The primers of monitored genes in qPCR are shown in Supplementary Table S3.

### Availability of data and materials

Raw sequence data generated in this study has been submitted to the National Center for Biotechnology Information Sequence Read Archive (SRA; http://www.ncbi.nlm.nih.gov/sra/) under accession no. GSE95785.

## Electronic supplementary material

Supplementary Information
