# Supplementary material for: A comparison of transcriptomic patterns measured in the skin of Chinese fine and coarse wool sheep breeds
Source: Sci Rep. 2017 Oct 30;7:14301. doi: 10.1038/s41598-017-14772-4 (PMC5662721; doi:10.1038/s41598-017-14772-4)
Supplement: Supplementary file 1 — Supplementary Information [file 41598_2017_14772_MOESM1_ESM.pdf]

Supplementary Information

A comparison of transcriptomic patterns measured in the skin of Chinese fine and coarse wool sheep breeds

Lichun Zhang[1, 2], Fuliang Sun[3], Haiguo Jin[1], Brian P. Dalrymple[4], Yang Cao[1], Tian Wei[1], Tony Vuocolo[2], Mingxin Zhang[1], Qinlin Piao[1], Aaron Ingham[2*]

[1] Branch of husbandry, Jilin academy of agricultural science (JAAS), 186 Dong Xinghua st, 136100 Gongzhuling, Jilin, China;

[2] CSIRO Agriculture, Queensland Bioscience Precinct, 306 Carmody Rd, 4067 Queensland, Australia;

[3] Department of Veterinary Medicine, College of Agriculture, Yanbian University, Yanji 133002, China.

[4] UWA Institute of Agriculture, The University of Western Australia, Crawley, Perth, Western Australia 6009.

Corresponding author:

Aaron Ingham, aaron.ingham@csiro.au

Supplementary Figures

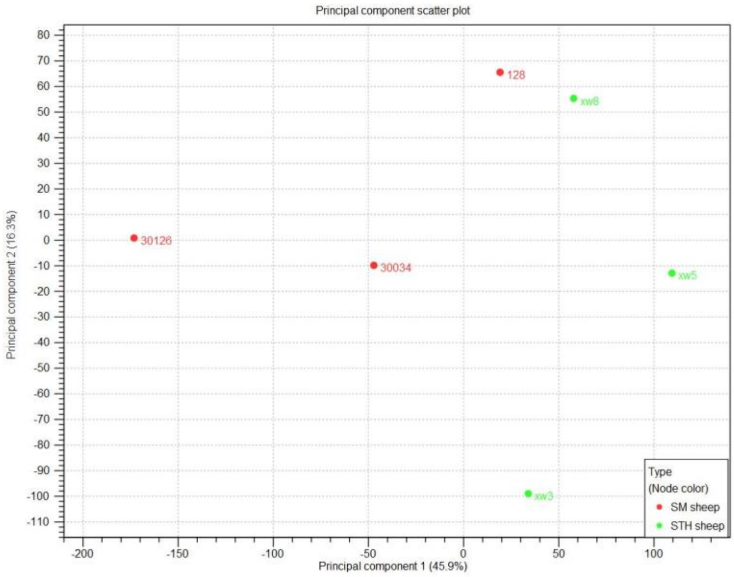

Figure S1. Principal component analysis (PCA) of RNA-seq.

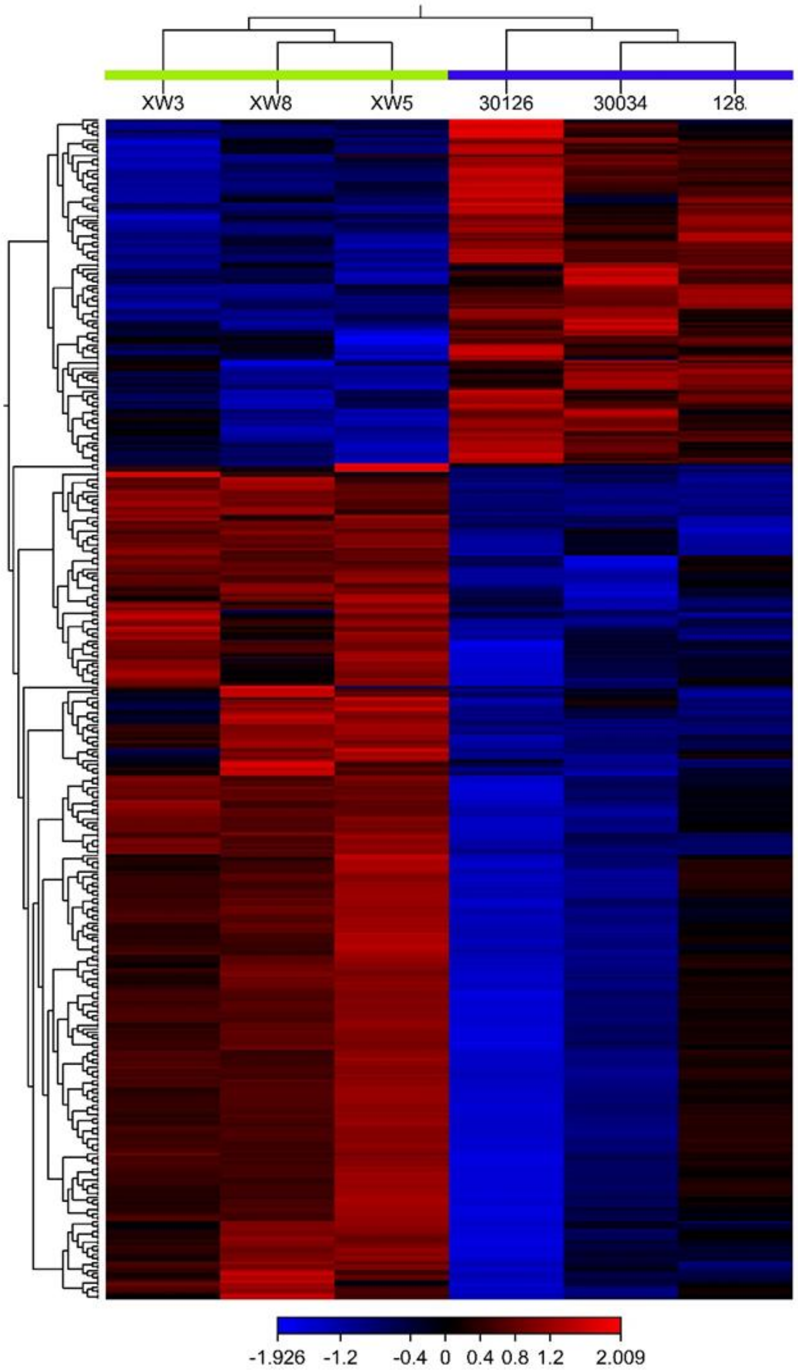

Figure S2. Heat map of differential expression genes (DEGs).

Supplementary tables

Table S1: statistic of reads mapping

		Super Merino sheep(SM)			Small Tail Han sheep(STH)		
		30034	128	30126	XW3	XW5	XW8
All reads		65,096,892	45,630,916	57,271,982	45,511,562	64,656,682	46,058,870
Reads Mapped in pairs		59,720,786	41,806,006	52,178,312	41,592,552	59,458,486	42,105,938
Rate of reads Mapped in pairs (%)		91.74	91.62	91.11	91.39	91.96	91.42
Reads mapped in broken pairs		3,188,098	2,297,024	2,960,971	2,443,128	3,220,482	2,481,568
Rate of reads mapped in broken pairs (%)		4.9	5.03	5.17	5.37	4.98	5.39
Un Mapped reads		2,188,008	1,527,886	2,132,699	1,475,882	1,977,714	1,471,364
Rate of un Mapped reads (%)		3.36	3.35	3.72	3.24	3.06	3.19
Counted fragments		62,908,884	44,103,030	55,139,283	44,035,680	62,678,968	44,587,506
unique fragments	Exon	19,688,226	13,140,754	18,234,734	13,856,288	18,729,808	13,758,250
	Exon-exon	21,473,634	13,614,794	21,131,829	14,565,841	19,848,431	13,570,958
	Intron	9,499,186	8,625,477	5,562,372	6,926,141	11,741,440	8,360,456
	Intergenic	6,162,754	4,735,410	5,038,200	4,365,490	6,111,983	4,793,455
	Total	56,823,800	40,116,435	49,967,135	39,713,760	56,431,662	40,483,119
Unique mapping rate (%)		87.29	87.92	87.25	87.26	87.28	87.89
Non-specifically		6,085,084	3,986,595	5,172,148	4,321,920	6,247,306	4,104,387
Uncounted fragments		2,188,008	1,527,886	2,132,699	1,475,882	1,977,714	1,471,364

Table S2: The DEGs list between SM and STH sheep

Name	Chromosome	Region	Identifier	Log_2 fold change	Fold change	P-value	FDR p-value	30034 - RPKM	128 - RPKM	30126 - RPKM	XW8 - RPKM	XW3 - RPKM	XW5 - RPKM
RPTN	NC_019458	complement(100922011..100928773)	105613036	-3.40764	-10.6121	0	0	1.773849	1.807308	1.891288	19.88783	10.12132	26.11188
DGAT2L6	NC_019484	59620107..59646677	101122168	2.384485	5.221573	0	0	3.022	4.521074	6.228749	0.768679	0.774258	1.013876
PLN	NC_019465	complement(19451607..19465519)	101102067	-3.32095	-9.99321	8.77E-15	8.3E-11	0.221624	0.853542	0.18964	2.704783	4.08427	5.583176
SPINK9	NC_019462	57172889..57181578	101109177	-4.84958	-28.8316	4.91E-14	3.48E-10	0.039666	0.141448	0.045255	0.559646	3.824948	2.020415
MX1	NC_019458	259561625..259596486	443146	2.479967	5.578846	7.12E-14	4.04E-10	26.74101	9.064094	10.8042	3.301182	3.109782	1.681123
FABP4	NC_019466	complement(57396171..57400688)	100137067	-1.83862	-3.57667	1.13E-12	5.33E-09	112.6266	47.73511	52.90771	217.4767	235.0566	284.4986
HNF4A	NC_019470	72275071..72305782	100534656	4.051835	16.58532	2.03E-12	8.24E-09	1.396821	1.316815	0.192752	0.042566	0.086198	0.042391
LOC101113331	NC_019462	complement(65941708..65952658)	101113331	-4.28682	-19.5191	4.82E-12	1.71E-08	0.023532	0.041958	0.026848	0.431623	0.571503	0.720365
PLIN4	NC_019462	17131002..17139777	101114356	1.778589	3.430904	1.75E-11	5.51E-08	28.70705	38.42604	68.45874	12.75663	13.36526	12.29618
SFRP2	NC_019474	3727931..3735374	100302355	-2.4773	-5.56854	3.85E-11	1.09E-07	1.149542	0.717375	1.741865	6.614303	6.56889	6.543973
LOC105613042	NC_019458	complement(100998306..101006564)	105613042	-2.23483	-4.70707	4.64E-11	1.2E-07	23.70618	37.58135	12.39749	102.1398	78.09701	157.4662
TFF2	NC_019458	complement(260517767..260521339)	101104197	-3.51961	-11.4685	2.54E-10	6E-07	0.193854	0.138257	0.387046	2.222268	4.119442	1.945648
SEMA3C	NC_019461	39918288..40126744	101114604	-1.42382	-2.68296	3.23E-10	7.05E-07	4.507295	6.786673	3.771591	12.73689	11.08365	15.52547
IFI6	NC_019459	238530640..238534368	101106828	2.086878	4.248277	5.18E-10	9.8E-07	107.4246	39.94699	58.05795	19.05649	20.68386	7.215009
LOC101116537	NC_019484	2851201..2857115	101116537	-2.51253	-5.7062	4.95E-10	9.8E-07	2.203476	0.953962	0.409624	7.399786	4.113341	8.125537
LOC101123419	NC_019477	26398840..26406039	101123419	1.555398	2.939149	7.38E-10	1.31E-06	20.77898	16.03478	10.22023	4.460779	6.894209	4.253015
NEXN	NC_019458	53628803..53681695	101122738	-1.66239	-3.16541	1.13E-09	1.89E-06	3.753157	5.16282	2.184588	9.122034	12.05364	13.10756
DES	NC_019459	220321994..220328992	101117308	-1.31222	-2.48324	1.32E-09	2.08E-06	25.07939	17.53844	24.13961	38.27432	66.86671	55.98275
MX2	NC_019458	259515453..259554045	780441	3.380272	10.4127	1.77E-09	2.64E-06	1.904283	0.710102	0.69265	0.239839	0.037583	0.028435
GPA33	NC_019458	complement(117076246..117128021)	101110729	4.206692	18.46462	2.15E-09	3.05E-06	0.224571	0.770524	0.941764	0.034254	0	0.060918
ISG15	NC_019469	complement(49466564..49467619)	443057	3.023443	8.131058	3.94E-09	5.33E-06	17.30319	4.832202	4.935341	0.808872	1.861382	0.575402
LOC101123672	NC_019477	complement(26412306..26427047)	101123672	1.448603	2.729437	5.25E-09	6.78E-06	40.17686	32.67987	21.91151	9.706301	14.97312	9.191553
DHCR24	NC_019458	complement(28996772..29028117)	101123162	1.171305	2.252153	6.03E-09	7.45E-06	146.5548	121.4866	195.1464	67.01196	63.16596	69.16908
LOC101110417	NC_019464	complement(83735106..83750552)	101110417	1.356416	2.560482	7.51E-09	8.89E-06	12.87038	13.40198	24.07558	6.629882	5.153734	7.253724
LOC101120001	NC_019469	4466942..4474363	101120001	-1.16416	-2.24103	8.84E-09	1E-05	24.82235	21.93606	22.26859	43.87454	52.71782	53.70583
LOC105607568	NC_019471	44478045..44480837	105607568	-13.5144	-11701.3	9.63E-09	1.05E-05	0	0	0	30.13397	2.397768	4.000862
NNAT	NC_019470	65917335..65920258	101122618	1.500463	2.829335	1.35E-08	1.42E-05	19.81333	10.00754	16.21021	5.136003	4.282653	6.270476
LOC105616741	NC_019470	66764519..66792369	105616741	-3.13764	-8.80084	1.5E-08	1.52E-05	0.20289	0.339735	0.412637	6.353716	0.579693	1.212953
COL6A5	NC_019458	complement(269653942..269821417)	101118494	-2.9969	-7.98281	1.58E-08	1.55E-05	0.60629	0.242246	0.170508	3.543881	0.332384	3.91358
TCHHL1	NC_019458	complement(100854244..100861502)	101120712	-1.71613	-3.28553	1.67E-08	1.58E-05	15.06256	14.21067	9.190952	67.4099	24.74404	30.36937
LOC101112509	NC_019472	complement(22032357..22044601)	101112509	-1.2022	-2.30091	1.81E-08	1.65E-05	49.19284	48.32623	40.46722	114.5762	79.9049	113.4139
LOC101123159	NC_019477	complement(26374549..26385517)	101123159	1.494389	2.817448	1.88E-08	1.67E-05	9.229788	8.100781	5.785552	2.184698	3.769216	2.065891
SENP6	NC_019465	2325109..2462063	101118793	-1.41844	-2.67297	2.93E-08	2.52E-05	5.519306	8.696814	4.189049	16.0735	13.31781	18.55251
LOC101107056	NC_019479	15694398..15743953	101107056	2.971038	7.841001	3.83E-08	3.2E-05	0.924384	0.590516	0.349389	0.064011	0.105321	0.062611
OAS1	NC_019474	complement(61037615..61063301)	105602911	1.853153	3.612888	5.12E-08	4.16E-05	25.31294	9.981787	8.151179	5.036113	4.148656	2.461911
CYP2J	NC_019458	complement(34592121..34659854)	768095	1.06774	2.096148	5.28E-08	4.16E-05	28.86155	31.03424	30.95961	15.04692	12.09288	14.9011
CLEC4F	NC_019460	complement(92668650..92679885)	101102383	3.306366	9.892711	6.24E-08	4.79E-05	1.806571	0.505022	0.673234	0.038426	0.181567	0.072892
ZNF175	NC_019471	56371604..56378089	101123658	-1.51864	-2.8652	7.78E-08	5.81E-05	1.895233	2.92618	1.53261	5.186385	5.348074	7.169774

LOC101119041	NC_019462	complement(7356284..7381259)	101119041	1.256835	2.38971	1.24E-07	8.65E-05	55.7409	44.67696	91.5842	26.86717	22.51152	28.36037
CES4A	NC_019471	33926704..33941237	101109991	1.286999	2.4402	1.19E-07	8.65E-05	86.13325	90.27051	153.1702	39.44071	34.1202	57.12631
LOC105602976	NC_019474	70405502..70413078	105602976	-7.61157	-195.574	1.25E-07	8.65E-05	0	0	0.014235	2.394177	0.03565	1.365008
OAS2	NC_019474	complement(61002833..61035319)	101109915	2.58478	5.999239	1.31E-07	8.84E-05	1.414001	0.406888	0.286955	0.121173	0.16651	0.053874
LRAT	NC_019474	complement(2473350..2480205)	101123410	1.154964	2.226788	1.49E-07	9.85E-05	18.91705	26.07835	33.56517	8.645095	13.02191	12.68456
SDS	NC_019474	60681920..60689944	101112348	1.546275	2.92062	1.6E-07	0.000104	5.805498	7.450087	5.046509	1.727359	1.749005	2.576473
GCNT1	NC_019459	complement(59816036..59859869)	101121316	-1.29418	-2.45238	2.3E-07	0.000143	0.966528	0.581535	0.651195	1.711618	1.696133	1.836353
KMO	NC_019469	complement(33248795..33317791)	101121026	-2.1719	-4.50617	2.31E-07	0.000143	0.136315	0.539672	0.199958	1.538625	1.21612	1.105689
CCL26	NC_019481	complement(33931038..33935665)	101117297	-1.54923	-2.92661	2.87E-07	0.000174	20.68752	6.964212	10.98517	43.7983	28.22387	37.01401
KERA	NC_019460	complement(127180428..127187605)	101112715	-1.85772	-3.62434	2.95E-07	0.000175	5.462605	12.08872	3.186306	36.55361	20.32506	16.75644
LOC101116409	NC_019465	complement(52736262..52744363)	101116409	-2.38739	-5.23209	3.28E-07	0.00019	6.020022	67.16197	22.3257	117.82	196.1543	179.8678
PCTP	NC_019468	5774689..5803850	101119229	1.302729	2.46695	3.74E-07	0.000213	18.95526	15.66719	33.8347	8.661184	10.67576	7.628854
LOC101113339	NC_019466	complement(57381426..57384616)	101113339	-1.11774	-2.17007	4.8E-07	0.000267	371.6341	387.0912	309.3177	868.6378	663.0838	720.6032
IL33	NC_019459	73691112..73822328	101116705	-1.09598	-2.13758	5.81E-07	0.000317	3.869642	4.435994	2.80992	6.449173	8.58142	8.111227
LOC105613724	NC_019458	105983948..106017666	105613724	-1.8865	-3.69736	6.05E-07	0.000324	0.26059	0.55193	0.486507	1.983184	1.004018	1.664385
RSAD2	NC_019460	16520599..16539212	101115013	2.112457	4.324271	6.56E-07	0.000345	5.279762	2.177746	0.959913	0.680237	0.648246	0.555056
CHORDC1	NC_019478	4577548..4601641	101108786	-1.09592	-2.13749	6.91E-07	0.000357	7.648893	9.798166	6.118868	13.57174	14.15424	21.24753
MYH11	NC_019481	complement(14090323..14217839)	101114409	-1.04813	-2.06784	7.13E-07	0.000361	33.76173	33.19641	35.94244	65.61665	74.52719	66.82163
LOC101107809	NC_019470	42366937..42380338	101107809	-3.65297	-12.5793	7.59E-07	0.000378	0.031108	0.11093	0	0.241395	0.63327	0.858598
FGL2	NC_019461	43979053..43982996	101118266	-1.13964	-2.20327	7.76E-07	0.00038	13.92572	16.01054	10.42585	32.27692	21.11682	32.91081
ADGRV1	NC_019462	87395891..87938369	101120749	1.406548	2.651021	8.38E-07	0.000403	0.550228	0.542825	0.428646	0.226854	0.146591	0.183271
LOC101112356	NC_019477	30658712..30661101	101112356	-2.4125	-5.32398	9.79E-07	0.000463	0.172927	0.151794	0.182118	0.938402	0.494084	1.181554
MCTP1	NC_019462	complement(91302996..91859584)	101122523	-1.43423	-2.70237	1.13E-06	0.000527	1.207007	1.153128	0.550834	2.562386	1.97695	3.090567
CENPE	NC_019463	21713052..21791689	101105752	-1.66488	-3.17087	1.16E-06	0.000529	0.947579	1.853216	0.730588	2.778343	2.38484	5.732334
TMC5	NC_019481	16969807..17042743	101116951	-4.34327	-20.298	1.3E-06	0.000584	0.013448	0	0	0.094872	0.076849	0.143976
NPHS2	NC_019469	complement(58781179..58802250)	101121618	4.287783	19.53221	1.53E-06	0.000679	8.785945	2.508834	1.173722	0	0.628168	0
LOC105605703	NC_019458	122945583..122945870	105605703	2.828772	7.104692	1.56E-06	0.000682	7.78242	2.834272	13.66489	0.934492	1.971255	0.443175
LOC105601854	NC_019459	complement(116421326..116421901)	105601854	1.113541	2.16376	1.71E-06	0.000735	79.45243	56.64608	104.6592	40.29997	41.98773	25.87036
PVRL3	NC_019458	174143229..174253856	101113983	-1.13911	-2.20246	1.89E-06	0.000791	0.696895	0.874446	0.567555	1.627567	1.256207	1.688919
BST-2B	NC_019462	5430867..5435031	100422801	1.793327	3.466133	1.89E-06	0.000791	7.851371	2.334613	2.990493	0.962896	1.742853	0.981991
LOC105607776	NC_019476	complement(51558740..51561200)	105607776	2.287718	4.88283	1.96E-06	0.000807	2.216635	7.406465	3.845432	1.392366	0.78082	0.533334
PGM5	NC_019459	complement(67752119..67958848)	101108630	-0.94128	-1.92023	2.16E-06	0.000876	7.860277	6.650842	7.671722	13.81315	15.01391	12.70094
PTX3	NC_019458	complement(228078905..228085039)	100034672	2.290341	4.891716	2.2E-06	0.000881	0.888701	1.315485	0.784358	0.23658	0.167681	0.185124
LOC101111178	NC_019460	133573147..133578596	101111178	-1.35227	-2.55313	2.36E-06	0.000931	30.30503	20.08512	26.714	93.56247	34.65669	61.70824
ESF1	NC_019470	complement(6775444..6835749)	101110781	-1.21431	-2.3203	2.59E-06	0.001006	3.482204	5.366001	2.786864	8.260799	6.979145	11.0183
BPIFB6	NC_019470	61537596..61559606	101106867	-2.4222	-5.35986	2.82E-06	0.001068	0.030435	0.040859	0.030638	0.212179	0.22251	0.100624
LOC101117112	NC_019475	complement(57521275..57527081)	101117112	1.292797	2.450026	2.82E-06	0.001068	43.26941	22.80494	32.96674	11.51139	18.30853	9.532105
KRT10	NC_019468	complement(40533235..40538366)	101106346	-1.33607	-2.52462	3.13E-06	0.001159	177.8067	143.9524	147.0425	498.6998	219.2858	425.5681
PLCB4	NC_019470	1483676..1963005	101106864	-1.42679	-2.68848	3.14E-06	0.001159	0.639903	0.722604	0.314336	0.917176	1.735813	1.744525
POSTN	NC_019467	24453914..24488210	101103329	-1.63148	-3.09831	3.23E-06	0.001176	126.7975	150.354	46.09436	389.9484	214.9	368.6576
LOC101108071	NC_019470	42564859..42586431	101108071	-4.27834	-19.4047	3.52E-06	0.001265	0.078075	0.022273	0.017815	0.110156	1.383054	0.940333
IVL	NC_019458	101659350..101662511	101123427	-1.0481	-2.0678	4.1E-06	0.001401	116.0902	85.56294	105.6915	258.9367	187.1719	170.0984

LOC101103522	NC_019458	complement(178432198..178434922)	101103522	-5.25334	-38.1428	3.97E-06	0.001401	0.035	0.016642	0.053243	0	0.016667	3.893438
TSKS	NC_019471	complement(55030780..55046115)	101108334	1.457623	2.746554	4.05E-06	0.001401	3.922507	4.381546	5.026962	2.059568	1.368928	1.30331
TMEM86A	NC_019478	complement(25344460..25348867)	101121641	0.948013	1.929214	4.03E-06	0.001401	22.14461	21.01594	29.69994	13.18445	9.445685	13.92863
FAM167A	NC_019459	complement(104095166..104126657)	101118417	0.898245	1.863798	4.68E-06	0.001545	14.77011	14.21379	16.87783	9.432944	8.050493	6.451042
CPM	NC_019460	150605403..150681068	101104474	-0.92644	-1.90058	4.68E-06	0.001545	9.224024	9.079279	7.629199	18.74792	12.47704	16.58152
CERS4	NC_019462	14521946..14550042	101102559	0.909402	1.878267	4.62E-06	0.001545	28.6837	28.43023	42.26005	16.3496	16.64213	18.34199
IL1F10	NC_019460	complement(59638718..59653603)	101111084	1.454229	2.740102	5.23E-06	0.001608	34.79744	22.49132	47.01356	8.561152	20.71471	7.891382
LARP7	NC_019463	complement(13069416..13088065)	101116230	-1.00472	-2.00655	5.27E-06	0.001608	9.338631	12.42988	7.514506	16.80293	17.75005	22.61592
TNFRSF11B	NC_019466	57987419..58016909	100037695	-3.01786	-8.09968	5.1E-06	0.001608	0.259527	0.154246	0.01645	1.485016	0.288366	1.606285
P2RX1	NC_019468	complement(24096822..24115485)	101101902	-2.58026	-5.98049	5.21E-06	0.001608	1.051389	0.249952	0.192783	5.73057	1.046038	1.821556
LOC101110259	NC_019470	73287609..73297034	101110259	-3.30629	-9.89222	5.23E-06	0.001608	0.471198	0.059305	0.221362	5.474966	0.316776	1.41878
LOC106991784	NC_019476	complement(51549903..51552443)	106991784	2.096956	4.278057	5.26E-06	0.001608	1.604952	4.419375	3.359678	0.984539	0.657939	0.504263
GDPD2	NC_019484	59864265..59875338	101101866	-2.7413	-6.68673	5.16E-06	0.001608	0.02988	0.074586	0.03409	0.474272	0.160072	0.254909
BCL6B	NC_019468	26398439..26404455	101114452	1.264749	2.402854	5.63E-06	0.001699	3.007352	2.618981	2.533349	1.135747	1.317954	0.880551
PPIG	NC_019459	complement(138770216..138812976)	101119793	-1.26527	-2.40372	6.96E-06	0.002059	5.114008	9.752035	4.367287	13.82083	11.55771	19.6916
CAPN8	NC_019469	complement(25003871..25079467)	101103336	-1.8655	-3.64393	6.95E-06	0.002059	0.569676	1.008394	0.814392	4.841927	1.529612	2.094176
PI3	NC_019470	72941426..72942784	641305	-2.75731	-6.76133	7.44E-06	0.002178	0.131644	1.690001	0.525678	7.847411	3.338155	4.294122
RDH12	NC_019464	76426876..76435627	101115797	1.122263	2.176881	7.8E-06	0.002261	24.4501	23.77784	50.09194	15.36832	13.46074	14.93614
LOC101109643	NC_019472	45647819..45655928	101109643	-1.01435	-2.02	8.37E-06	0.002401	3.51045	3.25155	2.891456	4.386005	8.557123	6.080738
FOXI3	NC_019460	complement(58843200..58851081)	101104229	1.353997	2.556193	9.29E-06	0.002629	3.052808	1.766536	3.017803	0.934792	1.048437	0.992349
LOC101110181	NC_019474	complement(61047492..61056487)	101110181	2.234179	4.704947	9.35E-06	0.002629	1.770172	0.872084	0.778643	0.32097	0.264056	0.128434
AADACL3	NC_019469	complement(52733414..52891787)	101112496	1.193837	2.287603	9.99E-06	0.00278	1.29441	1.41037	2.054886	0.700417	0.668166	0.652667
RGS5	NC_019458	complement(112746865..112800739)	101110554	-1.45455	-2.74071	1.02E-05	0.002801	12.85559	14.70454	4.627835	25.60727	20.30716	39.7709
PAQR7	NC_019459	240106357..240108379	101117571	1.184486	2.272823	1.03E-05	0.002812	25.77306	42.23248	36.87243	19.85576	8.722087	16.20668
LOC101116281	NC_019484	2824422..2831706	101116281	-1.86728	-3.64844	1.04E-05	0.002823	1.76192	1.657065	2.25318	11.9105	3.48516	4.663868
COL8A2	NC_019458	complement(10756459..10761140)	101117040	1.064565	2.091539	1.29E-05	0.003404	8.769399	8.809157	12.07806	3.32067	6.704392	3.822043
GPR155	NC_019459	134459450..134498177	101110651	-1.13309	-2.19328	1.31E-05	0.003404	2.34285	2.432956	1.260473	4.249012	3.468111	5.135728
LOC654331	NC_019460	complement(49916954..49919879)	654331	-2.128	-4.3711	1.3E-05	0.003404	3.068345	1.468315	1.084088	4.943614	3.167362	15.49734
LOC101117799	NC_019478	25460719..25461583	101117799	1.74697	3.356529	1.29E-05	0.003404	6.211387	3.722235	8.847799	1.840895	2.520289	1.106658
CSF3R	NC_019458	complement(11044427..11059493)	101118070	1.81344	3.514793	1.33E-05	0.003432	0.892703	1.111185	0.624547	0.201999	0.228592	0.287389
PCSK1N	NC_019484	52967697..52972144	101113828	1.1848	2.273318	1.37E-05	0.003494	8.576047	11.92402	15.31914	5.051859	5.940192	4.376206
ROCK1	NC_019480	35256061..35381753	101102442	-1.04666	-2.06574	1.39E-05	0.003529	7.728266	11.93547	5.956982	15.55432	15.63151	20.4128
MFAP4	NC_019468	33588943..33590925	101109814	0.854742	1.808435	1.46E-05	0.003659	75.51056	77.69203	95.7666	40.25242	53.70373	40.16776
ECI2	NC_019477	48837889..48853850	101121214	0.905422	1.873093	1.5E-05	0.003747	93.90281	92.56857	132.2056	61.29219	49.30864	54.43602
LOC101110773	NC_019467	29433047..29434884	101110773	2.894352	7.435101	1.58E-05	0.003889	1.159367	0.452192	7.233689	0.498396	0.155274	0.481811
STK32A	NC_019462	55848984..55985398	101114354	-1.41925	-2.67446	1.64E-05	0.004007	0.497719	0.462898	0.257925	1.450772	0.755312	0.957003
TPM2	NC_019459	52591784..52599233	101114931	-0.79725	-1.73778	1.71E-05	0.004147	29.84709	27.64916	31.9405	48.86623	57.1061	45.35718
FA2H	NC_019471	complement(1522608..1576330)	101116673	0.995434	1.99368	1.74E-05	0.004181	95.40045	101.79	167.3321	60.152	50.96412	66.08419
SLC45A4	NC_019466	15459151..15527205	101118622	0.891765	1.855445	1.95E-05	0.004648	3.927215	3.85453	4.347955	2.348399	2.163134	1.866934
KRT79	NC_019460	133267694..133278564	101108641	0.983165	1.976798	2.13E-05	0.005041	302.3749	244.0721	326.5227	152.7934	117.6596	157.0788
GSDMC	NC_019466	complement(23689942..23708037)	101103902	-2.69999	-6.49795	2.18E-05	0.005116	0.171078	0.477886	0.105725	1.820374	0.224034	2.711518
TMPRSS11F	NC_019463	complement(83611459..83709947)	101121519	-1.33403	-2.52106	2.27E-05	0.00529	2.1998	2.39333	0.835258	5.35737	3.452867	4.4966

ACTA1	NC_019482	complement(777575..780357)	101107662	-2.17756	-4.52387	2.3E-05	0.005298	0.53432	0.249167	0.820629	0.884354	3.963408	2.258562
CRYM	NC_019481	complement(19041179..19069773)	101102280	1.591921	3.014504	2.33E-05	0.005339	7.47647	2.523976	3.826839	1.12482	1.708373	1.60031
LOC105614079	NC_019458	complement(101088513..101101488)	105614079	-1.2899	-2.44511	2.43E-05	0.005495	42.66913	40.04631	18.69287	89.04661	49.61445	101.5196
LOC101119933	NC_019472	complement(77699373..77708234)	101119933	-4.64013	-24.9355	2.44E-05	0.005495	0.028285	0	0.016135	0.818096	0.323258	0.184525
LOC101111233	NC_019477	27710194..27713220	101111233	-3.16591	-8.97499	2.46E-05	0.005495	0.064408	0.128622	0	0.972356	0.62569	0.11636
KCND3	NC_019458	complement(88199539..88422824)	101115919	-1.27425	-2.41873	2.63E-05	0.005621	0.3309	0.60425	0.366584	0.868333	0.954576	1.2354
CEP83	NC_019460	complement(130261508..130408651)	101115779	-1.23941	-2.36102	2.59E-05	0.005621	1.60571	2.320045	1.18574	3.511906	3.422289	4.787859
KTN1	NC_019464	64891149..64996574	101119479	-1.11976	-2.17311	2.61E-05	0.005621	19.77176	30.61884	13.32362	41.39489	37.35443	56.18354
LRRCC1	NC_019466	complement(89825713..89871374)	101118707	-1.25369	-2.38451	2.6E-05	0.005621	4.761941	8.720838	3.218326	11.66656	10.8961	16.29859
SLC25A20	NC_019476	51004174..51042203	100147786	0.944859	1.925001	2.62E-05	0.005621	35.10807	33.56079	54.92813	21.56139	18.87392	21.80071
GRIA3	NC_019484	101413004..101724133	443273	-1.51186	-2.85178	2.63E-05	0.005621	0.466922	0.704332	0.212143	0.982346	1.174698	1.685841
LOC101106588	NC_019462	complement(15020344..15066298)	101106588	-1.85875	-3.62694	2.68E-05	0.005685	0.607297	0.23822	0.271374	0.978227	1.691777	1.29524
SCEL	NC_019467	53105195..53233117	101103405	-1.03428	-2.04809	2.75E-05	0.005774	12.39983	13.32248	7.021615	23.30898	16.22574	25.57415
SUMF2	NC_019481	27710881..27722110	101109064	0.949665	1.931424	2.77E-05	0.005791	10.53789	8.948005	13.46479	4.419919	6.26986	5.875857
DTNA	NC_019480	complement(22384594..22694486)	101111507	-1.21137	-2.31557	2.82E-05	0.005841	0.453394	0.462408	0.483658	1.052303	1.1335	0.973823
PSMC6	NC_019464	complement(11227530..11252435)	101116401	-0.84581	-1.79728	2.87E-05	0.005915	22.5631	27.87176	17.31896	37.14395	41.97388	39.58235
SYNPO2	NC_019463	complement(6402800..6607232)	101110330	-0.8952	-1.85986	3.07E-05	0.006218	6.209161	6.264974	3.854808	7.744031	11.31908	10.52893
HSPH1	NC_019467	30011966..30035540	101110772	-0.83916	-1.789	3.06E-05	0.006218	18.00129	16.90966	13.31715	25.66153	25.81567	32.28331
PTPRQ	NC_019460	116114949..116419066	101103961	-2.81265	-7.02575	3.22E-05	0.006491	0.010518	0.026254	0.003	0.103875	0.04132	0.122715
LOC101112888	NC_019459	29244838..29319035	101112888	-1.38969	-2.62023	3.47E-05	0.006894	0.70025	1.113916	0.541205	1.475154	1.57201	2.944067
NAA15	NC_019474	complement(17967495..18038756)	101105853	-0.87828	-1.83818	3.45E-05	0.006894	10.83277	15.39524	9.160386	19.48143	21.14077	22.80372
LOC101103408	NC_019468	complement(29145973..29150387)	101103408	-1.0725	-2.10307	3.58E-05	0.007054	3.552794	3.613588	2.159035	6.692168	5.485358	6.862071
SERPINA12	NC_019475	complement(57960537..57976249)	101118733	-1.36708	-2.57948	3.7E-05	0.007241	2.244519	2.286856	4.481385	9.873664	3.962312	8.608733
DNAJC2	NC_019461	complement(44518325..44549601)	101119299	-0.98117	-1.97407	3.8E-05	0.007337	11.77986	18.8704	10.25112	26.16766	21.43899	31.00844
ANKRD12	NC_019480	41743534..41841756	101106986	-1.38143	-2.60527	3.8E-05	0.007337	4.14277	9.352214	3.155321	12.96951	9.478512	19.87798
PLCB1	NC_019470	393916..1234909	101103585	-1.14685	-2.21429	3.89E-05	0.007457	0.764397	1.309227	0.665943	1.527697	1.864405	2.518951
LOC105601958	NC_019464	complement(24060801..24062553)	105601958	-3.83322	-14.2532	4.04E-05	0.007687	0.054407	0	0	0.230291	0.336811	0.345844
NUCB2	NC_019472	complement(34652411..34695603)	101114639	-0.98836	-1.98393	4.06E-05	0.007687	5.717365	5.877061	3.156485	8.654873	9.923523	9.911673
ZNF280D	NC_019464	50583804..50711863	101120087	-1.14715	-2.21475	4.09E-05	0.007692	0.942517	1.607154	0.631822	2.033522	2.076092	2.764694
LOC101122637	NC_019477	complement(29866379..29866882)	101122637	5.598832	48.46368	4.4E-05	0.008214	0.189238	0.134965	3.922246	0	0	0.063311
APCDD1L	NC_019470	56984375..57126334	101102919	1.220347	2.330027	4.61E-05	0.008562	5.507239	5.688024	9.580691	2.073924	4.540353	2.103318
CIDEA	NC_019480	43093842..43104646	101108619	1.130741	2.189712	4.65E-05	0.008572	12.39425	13.94259	21.63295	9.127011	4.404296	7.655209
DYNLT3	NC_019484	complement(35106936..35117796)	443256	-0.88229	-1.8433	4.75E-05	0.008704	17.52182	30.69752	18.11831	37.60436	39.84783	41.93131
ELOVL3	NC_019479	22007789..22010799	101106896	1.067598	2.095942	5.07E-05	0.009229	242.6808	214.6439	424.7585	120.9838	124.1556	162.3322
GLRX	NC_019462	complement(92397350..92397464)	100147797	1.2459	2.371665	5.11E-05	0.009245	103.1167	74.92337	126.3204	34.71435	61.21518	29.41143
ZC3H15	NC_019459	complement(121755246..121775342)	101116282	-0.8047	-1.74678	5.32E-05	0.009554	18.08634	19.58433	13.49626	27.23377	30.73864	29.03944
UACA	NC_019464	complement(17337918..17427151)	101107524	-0.94541	-1.92574	5.45E-05	0.009732	13.34563	17.95127	8.719595	25.15265	22.58811	27.35116
AKAP9	NC_019461	9170023..9338992	101109958	-1.17036	-2.25068	5.54E-05	0.009781	8.093117	15.10396	6.130873	20.73215	16.01839	27.61112
CCPG1	NC_019464	51881993..51922887	105601860	-0.92337	-1.89654	5.55E-05	0.009781	12.40026	19.11337	9.899867	24.27964	22.96009	29.32133
DIS3	NC_019467	complement(47921723..47946810)	101122118	-1.05799	-2.08202	5.63E-05	0.009816	2.470002	3.318588	1.527692	5.266217	3.979516	5.574891
AP1S2	NC_019484	complement(13264264..13291184)	101104633	-0.86102	-1.81632	5.6E-05	0.009816	7.680469	7.86017	5.277977	9.342298	13.86525	13.61599
SCN1A	NC_019459	142287237..142463962	101123531	-2.60439	-6.08133	5.69E-05	0.009853	0.003644	0.036383	0.020786	0.123385	0.119726	0.104229

MAPK6	NC_019464	complement(55285866..55318516)	101103324	-0.84905	-1.80131	5.74E-05	0.009885	15.96358	20.34464	11.71937	27.02037	27.83096	29.45557
KIF21A	NC_019460	147649692..147832474	101119971	-0.96252	-1.94871	6.06E-05	0.010373	9.151261	14.70738	7.586795	20.41456	16.92187	22.39273
LOC101120378	NC_019458	complement(101054250..101061849)	101120378	-1.1353	-2.19665	6.19E-05	0.010531	13.3065	18.3222	6.928412	31.75033	20.8461	29.87942
LOC101118931	NC_019484	124531794..124564053	101118931	-1.69697	-3.2422	6.23E-05	0.010537	0.18152	0.249675	0.140531	0.44819	0.61125	0.722239
GP5	NC_019458	191229239..191233282	101103118	-1.7598	-3.38651	6.3E-05	0.01054	0.224395	0.205765	0.164581	0.559706	0.629689	0.752069
MC4R	NC_019480	complement(59347550..59350098)	100147707	-9.38589	-669.014	6.31E-05	0.01054	0	0	0	0.985451	0.463264	0.425615
NEMF	NC_019464	complement(39845065..39900606)	101101894	-1.11285	-2.16273	6.65E-05	0.011045	5.496351	9.437899	3.613027	12.39244	11.20769	15.53805
BIRC3	NC_019472	complement(5944968..5963007)	101120189	-0.94683	-1.92763	6.73E-05	0.011103	5.117296	6.569414	3.632351	8.892525	8.388582	11.44238
C1H21orf91	NC_019458	137439516..137471918	101116190	-0.96628	-1.9538	6.88E-05	0.011232	7.557925	11.52749	5.358264	13.80848	14.82944	17.95478
EEA1	NC_019460	complement(128841461..129057440)	101114003	-1.19893	-2.29569	6.86E-05	0.011232	4.217864	7.736194	3.16461	9.320572	8.748399	15.76425
SSB	NC_019459	complement(138662944..138672175)	101118333	-0.85315	-1.80644	6.93E-05	0.011238	34.26092	48.4761	28.43894	65.74823	58.95781	70.9518
SH3BGR	NC_019458	257478482..257546633	101123341	-1.18897	-2.2799	7.25E-05	0.011697	4.650567	3.860232	3.192516	5.931321	9.477664	10.50875
SLCO1A2	NC_019460	193316539..193433818	101119972	-3.73169	-13.2847	7.51E-05	0.012052	0.003623	0.010337	0.008268	0.132921	0.093175	0.072735
CCDC78	NC_019481	complement(557880..562514)	101104626	-3.82697	-14.1916	7.67E-05	0.012228	0	0.036989	0.014793	0.512217	0.074091	0.130133
PAGE4	NC_019484	complement(52395293..52400657)	101103624	-3.35247	-10.2139	7.75E-05	0.012294	0.080081	0.057114	0.045682	0.677919	0.343207	0.864025
MASP1	NC_019458	198078891..198145602	101111065	0.846941	1.798683	7.95E-05	0.012336	2.830492	2.992122	2.73834	1.47807	1.644144	1.495717
SDPR	NC_019459	complement(193103107..193119396)	101118507	-0.90771	-1.87607	8E-05	0.012336	2.782557	4.246401	2.671827	5.687726	6.43181	5.624557
SGOL2	NC_019459	201766632..201797238	101104300	-1.32887	-2.51205	7.99E-05	0.012336	1.086275	2.711575	0.944546	3.21779	3.103682	5.313695
LOC106990171	NC_019460	59780815..59784833	106990171	-1.70752	-3.26599	8.04E-05	0.012336	0.308507	0.338505	0.171476	1.199796	0.553737	0.837611
KITLG	NC_019460	complement(124463931..124594572)	443371	-0.98679	-1.98177	7.91E-05	0.012336	6.123632	5.711066	2.977805	10.24374	7.529241	10.71511
CNFN	NC_019471	49862012..49883113	101104084	-1.23926	-2.36078	7.96E-05	0.012336	0.866781	1.015984	0.864228	2.631997	1.27596	2.367805
AWAT1	NC_019484	59680646..59690183	101122414	1.298007	2.45889	8.19E-05	0.012508	5.224966	9.641257	18.48518	5.226596	3.289942	4.632999
AZGP1	NC_019481	complement(36339966..36344405)	101123437	1.404088	2.646505	8.25E-05	0.012533	6.250174	5.403209	3.411091	1.393387	1.681422	2.416909
KRTAP6-1	NC_019458	complement(123206755..123207367)	100499198	1.414349	2.665395	8.56E-05	0.012731	602.4664	752.9085	1338.772	372.5284	481.3315	134.4275
NEB	NC_019459	156690312..156893984	101118671	-1.59938	-3.03013	8.53E-05	0.012731	0.27358	0.814197	0.276678	1.606888	0.511461	1.908524
LOC105614177	NC_019461	112594941..112595704	105614177	-2.00746	-4.02072	8.56E-05	0.012731	1.227571	0.534208	0.522238	1.555856	3.834349	3.612692
RSF1	NC_019478	17520308..17675988	101116780	-0.92573	-1.89964	8.51E-05	0.012731	4.65854	6.743131	3.16415	8.056218	8.079443	10.82895
LOC101113601	NC_019466	89620425..89627314	101113601	-4.32174	-19.9975	8.71E-05	0.012885	0.043551	0	0	0.40964	0.47699	0.247693
CEP162	NC_019465	52263598..52359023	101117339	-1.14535	-2.21199	8.83E-05	0.01293	1.928207	3.357724	1.360637	4.444046	3.8692	6.016148
SMC3	NC_019479	30010191..30048752	101119007	-1.08267	-2.11795	8.83E-05	0.01293	10.42612	20.09347	9.179463	24.38017	22.39673	35.2403
SENP7	NC_019458	complement(164047061..164186542)	101111506	-1.1728	-2.25449	8.97E-05	0.012932	2.117312	3.488926	1.183243	4.857268	4.213896	5.858838
PSIP1	NC_019459	complement(83426169..83462898)	100233239	-0.85357	-1.80697	8.95E-05	0.012932	28.28033	41.85463	22.60253	47.46479	51.96619	63.92586
RIF1	NC_019459	complement(156895567..156958974)	101118926	-1.35765	-2.56267	8.89E-05	0.012932	1.079588	2.546059	0.641563	3.188768	2.916631	4.593773
C1H21orf33	NC_019458	262070317..262078368	100302318	0.843891	1.794884	9.05E-05	0.012971	27.30259	27.18153	35.26057	16.20239	16.48801	15.8963
LOC443322	NC_019460	complement(150116581..150140615)	443322	-3.75087	-13.4624	9.09E-05	0.012972	0	0.046655	0.074633	0.599921	0.140178	0.869937
SEC62	NC_019458	complement(215261288..215294063)	101119522	-0.95271	-1.93551	9.3E-05	0.013196	22.74755	35.2011	18.10596	44.49292	44.79566	54.28679
GGCT	NC_019461	65734892..65744585	101102474	-0.82383	-1.7701	9.34E-05	0.013196	12.44344	11.16618	9.235026	19.25961	16.97776	20.17673
UGDH	NC_019463	complement(58490854..58525038)	101114271	-0.73848	-1.66841	9.39E-05	0.013196	15.43271	13.6121	12.32755	20.11365	23.34029	23.6188
GOLGA4	NC_019476	10729066..10833035	101109394	-1.13415	-2.19489	9.54E-05	0.013339	10.10154	18.93013	8.657313	22.14164	20.99589	37.49484
PRPF40A	NC_019459	155755311..155813748	101116380	-0.95827	-1.94298	9.58E-05	0.01334	17.32014	23.86225	11.76394	31.27302	31.50024	37.51037
ECM2	NC_019459	28627247..28667618	101108189	-1.25562	-2.3877	9.93E-05	0.013717	8.529021	21.05751	6.872242	25.2981	26.25186	33.71719
NRXN1	NC_019460	73066113..74278588	101104646	-2.12991	-4.37691	0.0001	0.013717	0.12138	0.084871	0.027154	0.537273	0.217602	0.241262

POF1B	NC_019484	complement(73024346..73123532)	101102041	-1.32381	-2.50327	9.99E-05	0.013717	7.733847	17.5503	5.186293	27.61003	15.68377	31.15836
PPP1R12A	NC_019460	complement(115484615..115655544)	641303	-0.89411	-1.85847	0.000102	0.013902	15.10852	17.90827	10.0787	22.90001	25.0151	30.05971
NIN	NC_019464	complement(40652482..40755712)	101104912	-1.08416	-2.12015	0.000102	0.013902	2.883808	4.455233	1.640081	6.387628	5.038065	7.133328
ACAD9	NC_019476	58731288..58745933	101111495	0.783707	1.721548	0.000104	0.013997	44.0111	57.77862	62.11002	30.65296	28.2077	33.65444
LOC101116002	NC_019458	63033440..63062397	101116002	-1.52527	-2.8784	0.000109	0.014473	15.58583	6.59724	4.446377	28.34674	9.476649	35.73076
LOC101113219	NC_019460	complement(42944753..42945271)	101113219	1.176064	2.259594	0.00011	0.014473	12.9557	14.41711	21.2459	5.790609	7.613374	7.469939
SRD5A2	NC_019460	91902135..91951099	100820756	-4.89345	-29.7219	0.000108	0.014473	0.015478	0	0	0.283896	0.044224	0.264093
DGKB	NC_019461	complement(22424489..23306033)	101102720	-2.00283	-4.00786	0.00011	0.014473	0.097873	0.081721	0.021788	0.208819	0.245538	0.330635
FAM185A	NC_019461	44034100..44124744	101103470	-1.3109	-2.48097	0.000109	0.014473	0.84087	1.81917	0.84184	2.703069	1.538359	4.204736
ZG16B	NC_019481	complement(2203223..2205025)	105604849	2.597875	6.053941	0.000109	0.014473	2.101582	2.965821	1.122334	0	0.734604	0.269271
CEP120	NC_019462	27773888..27864407	101110325	-1.00885	-2.01231	0.000114	0.014785	3.180518	4.330073	1.987135	5.407201	5.442036	7.752449
MDK	NC_019472	74497609..74500468	101114137	1.003939	2.005468	0.000113	0.014785	15.11714	14.99829	19.22647	5.744316	9.912998	8.279188
ATRX	NC_019484	64641762..64917198	101117394	-1.11588	-2.16728	0.000115	0.014964	6.894793	12.39274	5.062833	15.80889	13.43271	22.21818
TBC1D23	NC_019458	162845375..162900677	101108794	-0.84435	-1.79545	0.000118	0.01519	22.96368	26.74482	15.60353	39.1221	38.98652	36.17986
OGFOD3	NC_019468	49473464..49488072	101104767	0.960693	1.946244	0.00012	0.015459	10.52821	9.222805	13.51493	5.542405	6.587834	4.542633
MMRN1	NC_019463	complement(34599682..34705043)	101117586	-1.45789	-2.74706	0.000124	0.015858	0.338991	1.365286	0.546011	1.913139	2.103287	2.044741
CCDC112	NC_019464	complement(3862729..3889022)	101123303	-0.90402	-1.87127	0.000125	0.015873	0.992922	1.178608	1.008723	1.603386	2.040094	2.123215
DNAJB4	NC_019458	53715910..53759449	101101935	-0.95119	-1.93346	0.000127	0.016084	2.629226	2.997031	1.547901	3.93121	4.009741	5.542771
ROCK2	NC_019460	complement(19976250..20109888)	101103800	-1.06865	-2.09748	0.000127	0.016084	5.036542	8.495183	3.782517	10.13258	9.6846	15.57902
ARID4A	NC_019464	67459988..67518849	101122697	-0.96688	-1.95462	0.000134	0.016844	5.328285	8.431529	3.813218	10.62952	9.280654	13.55973
NCDN	NC_019458	10282519..10291279	101115750	0.79588	1.736136	0.000135	0.016854	9.239743	6.255373	9.164545	4.122894	4.521922	5.111831
CEBPE	NC_019464	21353016..21355895	101122954	-3.21402	-9.27931	0.000135	0.016854	0.056975	0.040635	0.032502	0.582801	0.38662	0.300216
USP15	NC_019460	complement(156829611..156958015)	101113139	-0.97559	-1.96644	0.000137	0.016908	2.184695	3.004969	1.263224	3.711466	3.872979	4.785673
WNT2	NC_019461	50996179..51050036	100126577	1.071799	2.102053	0.000137	0.016908	3.552263	4.038739	5.027856	1.9817	1.794201	2.036245
TRPM7	NC_019464	56637394..56727196	100037693	-1.22155	-2.33198	0.000138	0.016908	1.785781	4.430354	1.473804	4.874914	5.552496	7.129433
P2RY14	NC_019458	234738284..234800900	101117637	-1.84668	-3.59672	0.000141	0.017205	0.167805	0.099732	0.159542	0.286082	0.519402	0.691225
DMXL1	NC_019462	complement(32076336..32207850)	101115363	-1.02101	-2.02934	0.000141	0.017205	2.238905	3.63267	1.407313	4.274344	4.500327	5.645578
ELOVL4	NC_019465	complement(6777159..6818334)	101113417	0.912038	1.881702	0.000145	0.017622	123.4389	105.3535	156.3183	59.16935	56.2394	82.69368
LOC105614635	NC_019460	complement(85626353..85632212)	105614635	-1.85448	-3.61622	0.000146	0.017649	0.189884	0.174119	0.043328	0.405691	0.492154	0.536348
SKIV2L2	NC_019473	complement(23797292..23888623)	101115066	-0.81414	-1.75825	0.00015	0.018	8.964911	10.06239	6.106597	14.06002	14.23621	14.71718
ACTG2	NC_019460	95503479..95527600	101114256	-0.71102	-1.63696	0.000154	0.018474	37.06799	35.832	42.16174	65.97073	65.80868	51.52982
AMELX	NC_019484	8828662..8833506	101118759	-3.91741	-15.1097	0.000155	0.018474	0.021054	0.090096	0	0.445586	0.330858	0.781867
CEBPZ	NC_019460	86648954..86672368	101121334	-1.06933	-2.09846	0.000156	0.018497	8.868348	18.99164	7.398668	23.16031	20.94231	28.21375
KRAS	NC_019460	189342162..189386130	101114005	-0.78251	-1.72012	0.000164	0.01945	5.970923	7.897673	4.655145	9.172018	11.75792	10.17468
SCN7A	NC_019459	141970165..142053353	101109522	-2.40863	-5.30971	0.000167	0.019559	0.004374	0.168465	0.054897	0.339441	0.390562	0.458785
PLB1	NC_019460	35242956..35375830	101113054	0.840899	1.791166	0.000167	0.019559	6.538188	6.257304	9.883291	4.857597	3.441644	3.97279
SEMA3E	NC_019461	36930372..37203081	101113839	-0.98075	-1.97349	0.000167	0.019559	2.627204	3.554678	1.861201	4.579903	4.244361	6.608761
SMC4	NC_019458	complement(224944115..224976211)	101106049	-1.03296	-2.04622	0.000168	0.019607	5.892375	11.50555	4.94549	13.01083	12.79083	18.82481
STRA6	NC_019475	33342881..33365753	101120527	1.310591	2.480431	0.00017	0.019661	2.521069	5.356254	7.107513	2.087396	2.568489	1.2585
DENND4A	NC_019464	complement(12340676..12450311)	101122440	-1.08019	-2.11432	0.000171	0.019782	1.7276	3.495582	1.356325	4.315869	3.927992	5.343108
KRT1	NC_019460	133400378..133406299	101109430	-1.19132	-2.28361	0.000173	0.019845	223.0614	175.6465	159.829	614.146	211.3827	406.6143
ADGRL3	NC_019463	76563989..77502551	101111884	-0.98888	-1.98464	0.000173	0.01985	1.02	1.262086	0.757564	1.786775	1.358874	2.70589

ABCA10	NC_019468	60176870..60227471	101108240	0.800581	1.741803	0.000185	0.021143	6.777071	6.340044	4.776621	4.103223	3.028075	2.856523
GAPT	NC_019473	complement(20862169..20867361)	101110362	-1.44579	-2.72412	0.000187	0.02122	1.531769	0.69115	0.606313	2.194264	2.969792	2.353144
SPTSSB	NC_019458	223940729..223972223	101103775	-0.7151	-1.64159	0.000188	0.021263	33.84578	31.489	34.36993	51.50209	60.00217	47.82135
LOC101108887	NC_019459	complement(68014500..68066852)	101108887	-0.86872	-1.82604	0.000192	0.021332	7.868031	10.9861	5.881531	15.788	12.77813	15.41098
LOC101101923	NC_019474	complement(68982349..68999079)	101101923	1.3916	2.623694	0.00019	0.021332	2.032201	4.538962	5.891277	2.193803	1.03316	1.386385
CHGA	NC_019475	56428370..56441509	101114907	2.273685	4.835567	0.00019	0.021332	1.360469	0.408544	0.308619	0.179602	0.159121	0.079851
HTATIP2	NC_019478	complement(23843262..23856881)	101114318	0.788195	1.726913	0.000192	0.021332	17.33751	15.36493	24.0723	8.998984	11.53779	11.37612
RAB11FIP2	NC_019479	complement(37177703..37220788)	101105457	-0.9446	-1.92465	0.000192	0.021332	4.30207	6.103012	2.732438	7.737281	7.890145	9.03109
GPR141	NC_019461	complement(59640949..59707492)	101114515	-1.83673	-3.57199	0.000196	0.021608	0.500943	0.306235	0.163294	0.858239	1.086242	1.436517
UPF2	NC_019470	complement(15587287..15682170)	101116751	-1.09941	-2.14267	0.000196	0.021608	7.827832	13.91262	4.928797	18.91662	14.29463	22.52841
KIF3A	NC_019462	19098082..19177319	101122522	-0.98769	-1.98301	0.000206	0.022548	3.836048	6.950931	2.94712	7.921007	8.229724	10.44268
IQGAP2	NC_019464	7416632..7724494	101105506	-0.77699	-1.71356	0.000207	0.022548	3.543937	4.929169	3.60876	6.082057	6.677168	7.387288
TRIP11	NC_019475	complement(55529993..55598668)	101113176	-1.05221	-2.0737	0.000207	0.022548	4.453722	8.357364	3.489265	10.41707	8.584826	13.96375
IFITM3	NC_019478	complement(49640241..49641703)	101104700	0.864462	1.82066	0.000212	0.02301	255.1987	162.9026	234.0233	85.12135	156.7791	106.4858
LOC101105274	NC_019472	complement(77716562..77722885)	101105274	-3.15097	-8.88252	0.000217	0.023425	0.116515	0.142456	0.01899	1.737862	0.546915	0.167061
NAIP	NC_019473	complement(10129481..10176025)	101110450	-1.96037	-3.89162	0.000221	0.023767	0.229598	0.278724	0.072454	0.210218	0.844429	1.14975
COPS2	NC_019464	58021237..58060221	101109182	-1.00057	-2.00078	0.000224	0.02381	2.453769	4.789062	1.923051	5.491738	5.415852	7.011354
ACSS2	NC_019470	63633566..63677889	101114126	0.764356	1.698612	0.000223	0.02381	28.32556	24.71516	41.79756	20.56898	17.6873	15.9218
LOC105605177	NC_019483	complement(18890549..18901383)	105605177	1.579175	2.987989	0.000223	0.02381	0.310638	0.437227	0.575035	0.095783	0.144006	0.185827
FHL1	NC_019484	complement(93754680..93797838)	100307044	-0.76017	-1.69369	0.000225	0.023888	15.63874	13.98397	17.44268	18.6435	33.86779	25.14563
RAB27B	NC_019480	54245796..54425963	101120036	-0.85726	-1.81159	0.000226	0.023893	4.828125	6.278481	3.374234	7.792609	7.481984	10.25231
ZCCHC6	NC_019459	33325747..33384555	101114240	-0.89445	-1.8589	0.000228	0.02402	3.233939	5.07881	2.28595	6.20394	5.81692	7.197776
KIF20B	NC_019479	11064849..11148849	101122564	-1.07677	-2.10931	0.00023	0.02402	2.084411	3.709767	1.524161	4.190802	4.415893	6.449718
THOC2	NC_019484	complement(101814463..101918311)	101123613	-1.04484	-2.06314	0.00023	0.02402	3.611161	6.312638	2.361517	7.469562	7.265708	10.01009
ZNF644	NC_019458	complement(67428741..67523259)	101122642	-1.22601	-2.33919	0.000232	0.024092	1.201538	2.476166	0.700833	2.714404	2.939701	4.359321
LOC101123186	NC_019459	complement(165081477..165082011)	101123186	-1.15034	-2.21967	0.000234	0.024166	43.88486	28.26856	35.5599	61.95944	132.1358	39.72177
CEP350	NC_019469	59168497..59323473	101122613	-1.05821	-2.08234	0.000234	0.024166	2.673892	5.36021	2.040727	6.040053	5.875878	8.576146
PHAX	NC_019462	complement(24712253..24722176)	101107861	-0.84616	-1.79771	0.000242	0.024848	11.81335	16.68803	8.950074	20.40719	21.37544	23.84208
SASS6	NC_019458	complement(76529023..76570279)	101112610	-1.11919	-2.17224	0.000247	0.025129	1.79828	3.004808	1.118275	4.115296	3.590958	4.831516
TPM1	NC_019464	complement(43997072..44026561)	100145865	-0.82686	-1.77382	0.000247	0.025129	104.8097	82.75769	72.15723	150.3935	139.8778	156.7046
UBE2L6	NC_019472	complement(77909601..77918254)	101121201	0.940409	1.919072	0.000246	0.025129	28.97903	15.3117	21.69343	9.997036	14.14091	9.282268
FGA	NC_019474	2688286..2696532	101102090	3.921958	15.15748	0.000254	0.02567	0.023223	0.016563	0.9472	0.032765	0.016588	0.011654
ZNF184	NC_019477	30034270..30055258	101121720	-1.10408	-2.14962	0.000254	0.02567	2.109625	2.653511	1.227775	5.892796	2.877861	3.750664
TMEM45A	NC_019458	163178215..163287672	101110467	0.758908	1.692209	0.000256	0.025731	19.16399	16.65198	26.31482	11.38048	10.60556	13.57491
FILIP1L	NC_019458	complement(162381751..162687240)	101107834	-1.0495	-2.06981	0.000264	0.026441	1.734734	1.220886	0.894874	2.782777	1.856622	3.071342
VPS13A	NC_019459	complement(58940523..59203757)	101112545	-1.12786	-2.18534	0.000267	0.026699	0.844868	1.959425	0.736358	2.122856	2.165532	3.272785
PIK3CA	NC_019458	complement(205904440..205989643)	101110015	-1.12144	-2.17564	0.000268	0.026731	1.154864	2.483896	0.945081	2.706437	2.904471	4.132708
LOC101116991	NC_019464	24035721..24041006	101116991	-1.20378	-2.30342	0.00027	0.026758	4.359751	1.506688	2.244833	6.545705	5.902824	5.654168
MYOCD	NC_019468	30646505..30735045	101104413	-1.37442	-2.59264	0.000271	0.026787	0.220951	0.19825	0.162635	0.397219	0.539657	0.527578
ALG12	NC_019460	complement(223256185..223265150)	101112896	0.886875	1.849166	0.000272	0.026801	6.745587	7.395624	10.24108	4.413732	5.11978	3.328018
PKN2	NC_019458	65236597..65378319	101118744	-0.87924	-1.83941	0.000273	0.026839	3.402151	5.179129	2.652663	5.886789	6.498291	7.759202
LOC101111915	NC_019477	30644308..30646897	101111915	-1.51807	-2.86407	0.000274	0.026865	0.441897	0.560289	0.686223	1.359523	1.104755	2.199108

CLOCK	NC_019463	complement(70783114..70907400)	100171391	-1.10959	-2.15784	0.000279	0.02711	1.558995	2.490204	0.868844	2.84833	2.972288	4.526855
ZNF518A	NC_019479	16692692..16720962	101110983	-1.32928	-2.51277	0.000279	0.02711	1.229882	2.956203	0.704577	3.770786	2.940787	5.301763
PRKRIR	NC_019472	complement(53827717..53856272)	101108951	-0.81807	-1.76305	0.000283	0.027387	6.841738	6.965168	3.946964	8.835669	11.1219	10.5273
PLIN2	NC_019459	complement(87123150..87137944)	100125354	0.971787	1.961268	0.000285	0.027411	115.5424	87.96615	200.5364	64.65524	56.17786	78.30842
UHRF1BP1L	NC_019460	complement(168907108..168994369)	101104223	-0.97099	-1.96018	0.000285	0.027411	1.546055	2.699866	1.084438	3.031936	3.181018	3.990415
PCSK1	NC_019462	complement(92996990..93049213)	443029	-2.1404	-4.40883	0.000291	0.027909	0.016179	0.061541	0.055377	0.220664	0.146384	0.189449
TCN2	NC_019474	69070520..69087488	101112851	1.076875	2.109462	0.000296	0.028264	7.515156	9.291274	17.97039	3.446369	6.524535	6.063297
LOC101112166	NC_019474	complement(69009235..69018621)	101112166	1.129727	2.188173	0.000303	0.02884	6.325495	13.96437	16.98871	7.11912	4.074275	5.367035
ALDH1A1	NC_019459	63267948..63323060	443343	-0.89904	-1.86483	0.000312	0.029551	7.381928	15.58168	12.61134	21.07869	24.68687	19.10986
DMD	NC_019484	complement(28400851..31063619)	101114682	-0.71334	-1.63959	0.000312	0.029551	2.201951	2.885618	2.358511	3.965787	3.670207	4.232188
PPIP5K2	NC_019462	99323075..99399412	101114441	-1.05719	-2.08087	0.000317	0.029774	4.376817	6.842192	2.187879	8.813853	8.094134	10.32338
HBS1L	NC_019465	complement(59954272..60041246)	101109622	-0.84497	-1.79623	0.000316	0.029774	3.437779	5.371095	2.759809	6.060888	6.868834	7.351264
FITM2	NC_019470	complement(72197197..72203246)	101108768	0.712099	1.638186	0.000318	0.029787	30.45554	28.42479	39.27087	19.83662	18.64377	19.66307
ELOVL1	NC_019458	complement(17809090..17813902)	101110801	0.942678	1.922093	0.00032	0.029877	164.1384	113.9981	242.0041	89.22045	77.13698	95.34383
BST1	NC_019463	110325067..110345003	101116575	2.426226	5.374856	0.000329	0.030597	4.789934	3.438526	4.06294	0.176684	0.044724	1.955994
CD2AP	NC_019477	20565474..20653099	101117542	-0.84704	-1.79881	0.000337	0.031286	9.820728	15.63388	7.928132	18.14019	18.40369	22.04171
ZFP69B	NC_019458	14786586..14810741	101122155	-2.45136	-5.46931	0.000339	0.031323	0.064461	0.045974	0.088253	0.163707	0.221011	0.67285
VPS26A	NC_019482	25222758..25248048	101104294	-0.73868	-1.66865	0.00034	0.031323	16.70703	21.87329	13.65649	26.65869	29.44071	28.88224
DNTTIP2	NC_019458	complement(70139416..70153481)	101107316	-0.92763	-1.90215	0.000342	0.031448	7.590119	12.09219	4.959298	14.37505	14.05457	17.31253
LOC101116267	NC_019458	63087970..63121602	101116267	-1.58261	-2.99511	0.000345	0.031549	2.714824	0.840728	0.68701	4.838358	1.301294	6.023202
FKBP1B	NC_019460	31509116..31519653	101108378	1.058279	2.082446	0.000346	0.031549	8.66895	7.675109	9.529367	3.572571	5.099445	3.494419
LOC101117868	NC_019471	58293788..58319087	101117868	-1.48947	-2.80785	0.000352	0.032018	0.518481	0.269724	0.424516	1.144627	0.967261	1.236674
LOC101109901	NC_019471	46732368..46753949	101109901	-1.28473	-2.43637	0.000356	0.032159	1.271383	0.83782	0.746473	2.695953	1.99685	2.096895
TKT	NC_019476	47704693..47727998	101107229	0.691205	1.614632	0.000355	0.032159	101.6007	98.01815	129.8072	66.52884	70.31257	61.49121
TSPAN18	NC_019472	72883345..73090817	101111390	0.723072	1.650693	0.000358	0.032259	5.448648	4.754768	7.734516	3.638616	3.901738	3.022068
PMS1	NC_019459	complement(118231689..118431309)	101111691	-0.93892	-1.91709	0.00036	0.032352	2.446101	3.900542	1.7786	4.878074	4.63148	5.67232
CASP8AP2	NC_019465	complement(47453359..47486333)	101109100	-1.18049	-2.26654	0.000366	0.0328	1.995543	4.04223	1.309901	4.483633	4.207875	7.558751
KIAA1551	NC_019460	complement(182421795..182453328)	101106510	-1.36031	-2.5674	0.000372	0.033215	1.891417	4.586844	1.080411	4.80851	4.510664	9.639171
CFL2	NC_019475	complement(43945030..43949313)	101118388	-0.81529	-1.75965	0.000374	0.033266	5.180842	4.154048	3.960643	6.267578	9.180016	7.339091
UBE2V2	NC_019466	32333007..32352511	101111640	-0.70028	-1.62482	0.000376	0.033274	39.84978	43.08278	29.02332	53.74657	62.66612	60.70675
ANGPT4	NC_019470	59383921..59435376	101107460	0.905413	1.873081	0.000376	0.033274	2.663196	2.936756	2.024236	1.048248	1.879864	1.055459
SYN2	NC_019476	complement(56772726..56927614)	101107824	1.334994	2.522744	0.000377	0.033274	2.992263	2.049087	2.662412	1.460374	0.5377	0.950711
CRABP1	NC_019475	29780456..29787473	101122057	0.699076	1.623465	0.00038	0.033393	71.33307	93.4451	83.48722	49.93807	51.34177	47.63172
TMEM119	NC_019474	64006897..64018175	101102841	0.82579	1.772505	0.000386	0.033793	20.91205	14.68588	21.76957	9.228475	14.98316	7.344277
HIF1A	NC_019464	70743923..70770256	443519	-0.78563	-1.72385	0.000388	0.033855	25.09964	26.01499	17.7895	29.88131	46.42769	39.51359
LOC101123254	NC_019479	10140647..10162699	101123254	-3.45199	-10.9434	0.000389	0.033911	0.011675	0.033308	0.013321	0.131783	0.016679	0.474587
FUOM	NC_019479	50696035..50698729	101113117	1.173594	2.255729	0.000395	0.034304	12.89744	13.60451	18.25954	7.34001	5.93527	6.019128
CCDC88A	NC_019460	68406824..68525029	101122010	-1.25913	-2.39351	0.000402	0.034785	1.614912	3.369773	0.851149	3.851092	3.544096	6.250098
U2SURP	NC_019458	complement(243833635..243887311)	101104449	-0.84685	-1.79857	0.000407	0.034871	9.41109	12.51254	6.058389	15.59443	14.57572	18.85019
KRTAP1-1	NC_019468	complement(40771911..40779617)	100294612	1.011936	2.016615	0.000406	0.034871	715.7815	487.5961	1022.856	341.1229	494.678	238.6737
ESCO1	NC_019480	34875801..34912630	101123257	-1.19153	-2.28395	0.000404	0.034871	1.057338	2.252849	0.72509	2.457878	2.429232	4.110078
OSBPL8	NC_019460	complement(111994126..112164151)	101122935	-0.92383	-1.89715	0.000408	0.034876	3.914747	7.050557	2.885052	7.529826	8.065939	10.07326

LOC101121369	NC_019471	56077621..56102315	101121369	-1.57795	-2.98545	0.000412	0.035167	1.124505	4.119078	0.821093	6.32091	3.35428	8.026817
ZNF260	NC_019471	46046570..46062211	105607589	-1.2244	-2.33659	0.000414	0.0352	1.193484	3.13631	1.249405	3.488502	3.306107	5.924907
THAP2	NC_019460	107456173..107472620	101119806	-1.1025	-2.14726	0.000424	0.035879	3.191421	4.270312	1.619642	5.060581	4.944298	8.956492
KIAA1024	NC_019475	24591719..24627550	101113798	-2.88119	-7.3676	0.000425	0.035879	0.00419	0.011953	0.00956	0.04138	0.056862	0.077795
TINAGL1	NC_019459	complement(234989197..234999118)	101119187	0.90311	1.870093	0.00043	0.036267	12.76662	9.143811	12.15648	6.859731	6.852877	4.042919
HPGDS	NC_019463	30220444..30246506	101116145	-2.03032	-4.08497	0.000434	0.036443	0.285899	0.271873	0.195711	0.376486	0.544577	2.046897
RBPMS2	NC_019464	42382789..42424722	101122793	-1.20486	-2.30515	0.000439	0.036669	0.542491	0.768946	0.459334	1.150514	1.657225	1.167723
GNG4	NC_019482	complement(8289317..8357374)	101114927	0.926109	1.900144	0.000439	0.036669	51.58368	39.4848	93.90423	28.53975	36.08663	29.83031
SPDYA	NC_019460	35534268..35570523	101123454	-1.9042	-3.74302	0.000441	0.036699	0.135067	0.3056	0.053138	0.486275	0.505676	0.799352
SYT1	NC_019460	114742194..115237316	101102712	-2.65561	-6.30113	0.000451	0.036699	0.056645	0.080799	0.04847	0.049951	0.515881	0.582741
SYNE2	NC_019464	72944183..73267530	101108755	-1.08807	-2.12589	0.000446	0.036699	6.532024	9.854788	4.832796	10.43991	11.52611	21.92423
NSRP1	NC_019468	20828779..20871165	101111561	-0.96662	-1.95426	0.000449	0.036699	11.07428	17.47394	7.17087	21.99455	18.27536	27.74464
ATP5G1	NC_019468	complement(37125809..37128499)	443410	0.798645	1.739466	0.000449	0.036699	31.59354	26.86929	47.09979	23.06424	20.41477	15.48347
SLC2A5	NC_019469	42437997..42465480	443507	-1.97853	-3.9409	0.000445	0.036699	0.089723	0.127982	0.272977	0.316479	0.747705	0.817978
MBOAT7	NC_019471	60146607..60164652	101107304	0.725004	1.652905	0.000445	0.036699	27.5506	30.88562	47.99231	19.51429	22.26317	20.80771
LY6G6D	NC_019477	complement(26643312..26645207)	101119341	1.092064	2.131788	0.000451	0.036699	305.4263	220.1353	602.8226	213.7499	197.0688	103.0718
P4HA1	NC_019482	complement(28841341..28936893)	101116374	-0.76857	-1.70358	0.000444	0.036699	6.995322	6.806103	4.784919	8.71981	9.978018	12.05377
LOC101115634	NC_019468	40898198..40899119	101115634	1.316145	2.49	0.000453	0.036755	1052.914	1210.599	948.0492	501.7667	637.4781	125.7784
SYTL2	NC_019478	9144837..9268661	101103681	-0.76906	-1.70416	0.000456	0.036922	3.606951	5.239577	3.018095	7.222955	6.368691	6.127315
IFT74	NC_019459	94644224..94757000	101110029	-0.87129	-1.8293	0.000462	0.0372	1.388603	2.13461	1.4361	2.497617	2.634524	3.687781
SLMAP	NC_019476	complement(43338987..43496457)	101123588	-0.69876	-1.62311	0.000462	0.0372	7.624092	9.207122	6.509897	11.36794	12.66788	12.85727
NBN	NC_019466	86231878..86286945	101115286	-0.96506	-1.95215	0.000468	0.037575	4.836513	8.209617	3.323074	9.030263	9.373737	12.76669
BST-2A	NC_019462	5441157..5449951	100422799	1.781027	3.436708	0.000471	0.037642	3.446672	0.915032	2.319707	0.506236	1.087291	0.305555
CHD1	NC_019462	complement(95292016..95363422)	101111801	-0.97406	-1.96436	0.00049	0.038614	6.095949	10.00123	3.742948	11.89745	11.0111	15.12157
HMGCR	NC_019464	6519416..6540790	101108919	0.758713	1.69198	0.000491	0.038614	100.5913	92.33684	113.6624	54.44195	54.36686	66.92344
LOC101114537	NC_019468	complement(40877006..40878061)	101114537	1.414584	2.665828	0.000487	0.038614	51.25544	53.1808	67.77516	31.4088	27.38054	4.335221
LOC101116068	NC_019468	40915453..40917620	101116068	1.297274	2.457641	0.00049	0.038614	285.8715	321.5383	310.8699	130.5323	198.6501	37.4426
PIK3C2A	NC_019472	34787560..34912953	101114899	-1.23782	-2.35842	0.000488	0.038614	1.87559	4.659501	1.093386	5.231527	5.108566	7.282359
DCLRE1A	NC_019479	complement(33197599..33221820)	101123252	-0.9431	-1.92266	0.000489	0.038614	2.179756	3.618001	1.689449	5.601869	4.086771	4.350191
BRWD1	NC_019458	complement(257197464..257312852)	101117901	-0.97653	-1.96773	0.000498	0.039055	1.350546	2.223556	0.86009	2.447003	2.443876	3.618497
GLS	NC_019459	192096683..192167498	101122583	-1.02099	-2.02931	0.000501	0.039222	0.827191	1.2451	0.467964	1.56848	1.545697	1.912729
LOC101122689	NC_019461	82563982..82597586	101122689	1.025332	2.035428	0.000505	0.03939	5.820338	4.411526	3.579564	2.187053	2.874539	1.570748
ZNF654	NC_019458	154490640..154552634	101102449	-1.0828	-2.11814	0.000526	0.039425	0.526658	1.035968	0.40704	1.130579	1.296436	1.645449
ADRA1A	NC_019459	38670865..38783309	100169940	-1.35196	-2.55259	0.000525	0.039425	0.358126	0.354178	0.177055	0.471597	0.746957	0.984864
CWC22	NC_019459	128927606..128994278	101121571	-0.95146	-1.93383	0.000526	0.039425	1.993197	3.099884	1.574575	4.266179	3.335701	4.943664
ZNF638	NC_019460	92957494..93093462	101108732	-1.1569	-2.22978	0.000509	0.039425	6.569189	13.85001	4.412625	15.85499	14.21928	24.01105
RAD50	NC_019462	complement(19252566..19387745)	101123035	-1.04081	-2.05738	0.000508	0.039425	2.90435	4.789466	1.713373	5.510338	5.046413	8.306577
SQLE	NC_019466	complement(27905527..27927180)	100125351	0.691732	1.615222	0.000517	0.039425	92.60392	68.3367	93.59956	46.79514	55.13923	51.02637
RCBTB1	NC_019467	complement(19400817..19454389)	101116583	0.681584	1.6039	0.000518	0.039425	5.845129	5.938894	5.366313	3.146892	3.141095	4.07994
ITGBL1	NC_019467	77266839..77400676	101112990	-0.84773	-1.79966	0.000511	0.039425	2.723381	3.42376	1.935373	5.515368	3.808164	4.818145
KRT36	NC_019468	complement(41124768..41128793)	101117175	0.799185	1.740118	0.000521	0.039425	37.99463	40.98636	61.96704	34.51684	26.15709	18.08735
MYBPC2	NC_019471	55508132..55533426	101111044	-1.50977	-2.84765	0.00052	0.039425	0.202086	0.453852	0.363012	0.825042	0.687961	1.286012

CLGN	NC_019474	16944599..16989922	101115069	-1.61402	-3.06103	0.000524	0.039425	0.377013	0.420866	0.168315	0.474112	1.328928	1.085831
MPHOSPH9	NC_019474	51897489..51956585	101118475	-0.97042	-1.95942	0.00052	0.039425	1.424467	2.12471	0.933527	2.807412	2.33252	3.409983
MIPOL1	NC_019475	46262248..46572666	101121886	-1.56488	-2.95853	0.000521	0.039425	0.110758	0.243299	0.080874	0.368796	0.427215	0.451327
CDH19	NC_019480	10267958..10347258	101113295	-1.02382	-2.0333	0.000517	0.039425	0.64775	0.625906	0.533409	0.998675	1.238801	1.318603
LOC101117055	NC_019484	complement(2068580..2091239)	101117055	-0.89296	-1.85698	0.000519	0.039425	8.5494	9.806631	4.966708	13.13271	11.73519	17.21639
LOC105604204	NC_019479	1060093..1061183	105604204	-1.89764	-3.72603	0.000528	0.039448	0.364323	0.683781	0.175015	1.379757	1.616191	1.347168
HAP1	NC_019468	complement(41365627..41376261)	101108406	-1.30268	-2.46687	0.000533	0.039536	0.591778	0.986497	0.500272	2.655726	1.018563	1.313127
LOC105601907	NC_019471	complement(57665511..57935993)	105601907	-1.17485	-2.2577	0.000532	0.039536	0.249248	0.452492	0.213708	0.609556	0.574034	0.823263
IL5RA	NC_019476	23093898..23134747	101111837	-1.69211	-3.23128	0.000532	0.039536	0.169066	0.085745	0.077156	0.286245	0.343504	0.426102
CD200	NC_019458	175281088..175322456	101120715	0.834456	1.783185	0.00054	0.039789	5.583884	6.156745	5.358331	3.076222	3.006117	3.180685
CEP290	NC_019460	complement(124072063..124163568)	101109526	-1.12358	-2.17887	0.000539	0.039789	1.918747	3.77539	1.472995	4.197564	3.59891	7.418031
SFRP4	NC_019461	complement(49700370..49710532)	101107858	-2.15028	-4.43914	0.000543	0.039964	0.312227	0.314373	0.39813	3.23914	0.918325	0.276505
SERHL2	NC_019460	217199128..217219992	101106160	0.827181	1.774216	0.000546	0.04006	6.831127	7.730956	8.218443	4.018926	3.710598	4.675066
IGFBP4	NC_019468	40200887..40211101	443470	0.717624	1.644471	0.000547	0.04006	57.63069	45.05654	60.73306	28.05641	39.25096	29.40358
DTWD1	NC_019464	complement(57612456..57652122)	101108491	-0.99618	-1.99471	0.00055	0.04013	0.57896	0.820037	0.479761	1.028956	1.448143	1.174401
CWF19L2	NC_019472	15517979..15673564	101103667	-1.0068	-2.00944	0.000552	0.040197	3.303559	6.242339	2.480214	7.373251	6.936746	9.275321
CREB1	NC_019459	208576933..208633135	443118	-0.95048	-1.93252	0.00057	0.041321	2.054795	2.998118	1.302926	3.638347	3.14012	5.172263
CASP14	NC_019462	complement(7856497..7865023)	101120827	-0.91873	-1.89045	0.000571	0.041321	20.40472	16.23071	16.31059	33.08645	18.24775	45.26972
AHCTF1	NC_019469	28490710..28583117	101114886	-0.93294	-1.90916	0.000572	0.041321	1.981126	3.613925	1.471508	3.696956	4.235625	5.249275
JAK2	NC_019459	72812001..72927328	101113909	-0.94558	-1.92596	0.000592	0.042646	4.571208	7.399139	3.200231	8.72886	8.019022	11.72812
PIBF1	NC_019467	47946975..48174554	101123131	-1.37031	-2.58527	0.000593	0.042647	0.697902	1.489124	0.51328	2.213485	1.722116	2.853947
SLCO4C1	NC_019462	complement(98474880..98565923)	101104490	-1.64191	-3.1208	0.0006	0.042664	0.08688	0.153839	0.061524	0.249386	0.299589	0.348794
ANKRD6	NC_019465	complement(47648884..47832575)	101109885	-0.94619	-1.92678	0.000599	0.042664	0.464776	0.503662	0.489045	0.90601	0.755476	1.049995
AARD	NC_019466	complement(60074300..60080185)	101120843	2.867811	7.299567	0.000596	0.042664	0.752969	0.19094	0.209995	0	0.11952	0.033588
PDLIM3	NC_019483	complement(14429636..14458861)	101113652	-0.83204	-1.7802	0.000599	0.042664	5.401271	4.996891	5.568697	7.600018	12.91965	7.245274
TMF1	NC_019476	32492363..32526717	101116869	-0.86753	-1.82454	0.000611	0.043367	3.518209	5.437234	2.524711	7.178405	5.620967	7.606096
TLCD1	NC_019468	complement(19739970..19741303)	101120761	0.845097	1.796386	0.000614	0.043465	20.22636	20.74391	31.88705	16.16533	10.69714	12.45922
BDP1	NC_019473	complement(9995641..10078636)	101117279	-1.06229	-2.08825	0.000618	0.043629	4.636274	8.305598	2.798985	9.270875	8.543741	14.25461
STAG2	NC_019484	102120089..102248891	101116633	-0.98556	-1.98008	0.000628	0.044219	11.96727	18.70454	6.804648	21.30751	23.49587	27.67993
RPAP3	NC_019460	138506362..138540448	101110665	-0.89488	-1.85945	0.000633	0.044504	3.714475	6.316378	3.503805	7.552221	7.106173	9.843921
RLF	NC_019458	14498322..14583180	101121469	-0.84784	-1.7998	0.000637	0.044626	3.765022	5.347836	2.522535	6.785554	6.058284	7.55994
LOC101118574	NC_019478	complement(47524510..47525448)	101118574	-2.22086	-4.6617	0.000645	0.044967	0.203144	0.096589	0.212455	0.692659	0.798075	0.90051
PTEN	NC_019479	9230176..9331523	101119351	-0.81577	-1.76024	0.000643	0.044967	6.907383	9.286893	4.701818	10.87835	10.29486	14.63155
PRKCI	NC_019458	complement(214981545..215051409)	101118322	-0.81184	-1.75545	0.00065	0.045207	6.387093	8.477239	4.002054	9.291107	11.58864	11.44295
UFL1	NC_019465	complement(40276240..40343771)	101106268	-0.86505	-1.82141	0.000651	0.045207	4.473396	6.706226	3.114286	7.832345	7.562612	9.972831
PHOSPHO2	NC_019459	complement(138737714..138743344)	101119113	-0.89561	-1.8604	0.000665	0.045944	5.677966	6.098912	4.156961	6.843481	12.10154	9.955585
KIAA1033	NC_019460	173505129..173563236	101114433	-0.97467	-1.96519	0.000664	0.045944	2.702629	4.513888	1.683603	4.812404	5.464879	6.801564
PM20D2	NC_019465	complement(48049475..48066814)	101111105	-1.08073	-2.11511	0.000667	0.045976	0.628656	0.792635	0.416255	1.168115	1.094548	1.504167
ATP13A5	NC_019458	192134764..192252365	101107321	-1.54791	-2.92393	0.000674	0.046316	0.139148	0.174853	0.226793	0.387976	0.638953	0.508756
IQCB1	NC_019458	complement(184364855..184405924)	101110893	-0.91767	-1.88906	0.000684	0.04692	2.80749	2.635042	1.71045	4.586966	3.489351	5.021298
FAM171B	NC_019459	complement(121473424..121555430)	101116019	-0.90816	-1.87665	0.000689	0.047019	1.277448	1.984389	1.038058	1.88546	2.962277	3.029016
PDS5A	NC_019463	complement(58786702..58908657)	101114956	-0.82161	-1.76738	0.000688	0.047019	7.960882	11.40368	6.079583	13.5966	12.92754	17.28088

SLC35A3	NC_019458	76426736..76469420	101111921	-0.8359	-1.78497	0.000691	0.047042	2.213309	2.993379	1.587258	3.499491	3.67049	4.633263
CHI3L1	NC_019469	complement(352222..365563)	443279	0.702419	1.627231	0.000697	0.047329	25.88241	18.31032	26.87388	16.2727	12.58395	13.4573
SLC22A31	NC_019471	complement(13755718..13760962)	101109903	1.178024	2.262666	0.0007	0.047444	1.900698	2.491031	3.706876	1.329395	1.12558	1.027207
ZMYM1	NC_019458	9846238..9874293	101108698	-1.03933	-2.05528	0.000712	0.047793	1.280101	2.855159	1.063399	3.394233	2.85225	4.190074
FAM135A	NC_019466	complement(3641869..3802085)	101108058	-0.71028	-1.63613	0.000712	0.047793	3.672262	4.570022	2.705474	5.968457	5.246153	6.216082
MEIS3	NC_019471	complement(53328078..53338975)	101118811	0.825664	1.772351	0.000709	0.047793	6.568276	8.093961	8.336302	4.688454	3.789437	4.115355
SLC4A7	NC_019476	complement(1657846..1751785)	101102429	-0.70326	-1.62818	0.000708	0.047793	1.861885	2.351689	1.88285	3.077021	3.475964	3.103635
DSC1	NC_019480	26209584..26245539	101115748	-0.88781	-1.85036	0.000727	0.04871	48.15889	72.09196	40.07024	106.1051	80.70963	102.2171
ODF2L	NC_019458	complement(62743115..62780496)	101114828	-1.22892	-2.34392	0.000732	0.048795	0.659491	1.643527	0.536207	1.946524	1.884283	2.666661
MPDZ	NC_019459	complement(81070699..81238226)	101120218	-0.78095	-1.71827	0.000734	0.048795	1.68014	2.179141	1.20688	2.202356	3.107528	3.17936
SYNE1	NC_019465	complement(75665861..76158875)	101106269	-0.81395	-1.75802	0.000731	0.048795	1.518716	2.074461	0.984152	2.183494	2.417034	3.23911
HSD17B14	NC_019471	complement(54343584..54362364)	101123575	0.922387	1.895249	0.000736	0.048859	13.25603	10.89643	18.60584	8.955273	6.151987	6.765081
GOLGB1	NC_019458	complement(184271100..184347245)	101110468	-1.01417	-2.01974	0.000741	0.049021	6.599495	11.46783	4.644094	13.05186	11.12825	20.51229
LTN1	NC_019458	125198163..125255736	101109928	-0.94584	-1.92631	0.000745	0.04921	3.289111	4.662471	1.806364	5.292052	5.676485	7.358956
SLITRK6	NC_019467	complement(61179847..61186544)	101105831	-1.33188	-2.5173	0.00075	0.049382	1.022357	1.970531	0.438974	2.347216	2.067773	4.008807
CD24	NC_019465	30086266..30090226	101119731	-0.69002	-1.61331	0.000752	0.049397	37.97428	51.11952	39.02748	64.31847	71.24079	65.91766
CARNMT1	NC_019459	61309512..61348644	101122760	-0.80444	-1.74646	0.000756	0.049491	2.697171	3.853286	2.325979	4.627299	5.240042	5.227728
BRWD3	NC_019484	complement(67823466..67987104)	101120385	-0.77893	-1.71586	0.000756	0.049491	2.125818	3.261036	1.740264	3.494403	3.860316	4.569786
PRRG4	NC_019472	61598804..61617301	101122380	-0.71094	-1.63687	0.000765	0.049944	5.316812	7.310165	5.105111	9.142494	8.283065	10.79914

Table S3: qPCR primers list

Target gene	Forward primer(5'-3')	Reverse primer(5'-3')	Annealing temp. (℃)	Product size (bp)	Accession number
GAPDH	GGCGTGAACCACGAGAAGTA	GGCGTGGACAGTGGTCATAA	64	141	NM_001190390
CD200	CCTTGGAAGATGAGGCGTGT	ACAAAGAGAGTGAGGCAGGC	64	88	XM_012190412
CD24	TCCTCCCAGACTACCTCACC	AGAGACTGGCTGTTGACTGC	64	88	XM_004011234
FOXI3	CAGCTTCCCCTTCTACCAGC	TGAAGCAGTCGTTGAGGGAC	64	80	XM_012175964
TGFB1	GAGCCCTGGACACCAACTAC	GTAGCCCTTGGGTTCGTGAA	64	119	NM_001009400
RPTN	AAAGGCCACCCTGTCACAAA	GTGGCTCTGCTTCTCACCAT	64	104	XM_012181345
LOC101116068	TTGCCCTCCAACTTGCTATC	CCCACTGAAGGTTTGTCCAC	64	133	XM_012186088
KRT1	GATCTCGGCTGGATTCAGAG	TCTTGATGCCCACAAACTCA	60	120	XM_015094647
KRT10	TCGCTATTGTGTGCAGCTCT	TGTTGGTACTCGGCATTCTG	64	108	XM_015098774
FABP4	AATTGGGCCAGGAATTTGAT	GGTGGTTGATTTCCCATCC	60	116	NM_001114667
ELOVL3	CTTCGGGACAGTGAGGATGT	CAGGAAGGACCAGAATTGGA	62	121	XM_012102841
